# Identification and Characterization of Novel *Salmonella* Mobile Elements Involved in the Dissemination of Genes Linked to Virulence and Transmission

**DOI:** 10.1371/journal.pone.0041247

**Published:** 2012-07-20

**Authors:** Andrea I. Moreno Switt, Henk C. den Bakker, Craig A. Cummings, Lorraine D. Rodriguez-Rivera, Gregory Govoni, Matthew L. Raneiri, Lovorka Degoricija, Stephanie Brown, Karin Hoelzer, Joseph E. Peters, Elena Bolchacova, Manohar R. Furtado, Martin Wiedmann

**Affiliations:** 1 Department of Food Science, Cornell University, Ithaca, New York, United States of America; 2 Life Technologies Corporation, Foster City, California, United States of America; 3 Department of Microbiology, Cornell University, Ithaca, New York, United States of America; Institut National de la Recherche Agronomique, France

## Abstract

The genetic diversity represented by >2,500 different *Salmonella* serovars provides a yet largely uncharacterized reservoir of mobile elements that can contribute to the frequent emergence of new pathogenic strains of this important zoonotic pathogen. Currently, our understanding of *Salmonella* mobile elements is skewed by the fact that most studies have focused on highly virulent or common serovars. To gain a more global picture of mobile elements in *Salmonella*, we used prediction algorithms to screen for mobile elements in 16 sequenced *Salmonella* genomes representing serovars for which no prior genome scale mobile element data were available. From these results, selected mobile elements underwent further analyses in the form of validation studies, comparative analyses, and PCR-based population screens. Through this analysis we identified a novel plasmid that has two cointegrated replicons (IncI1-IncFIB); this plasmid type was found in four genomes representing different *Salmonella* serovars and contained a virulence gene array that had not been previously identified. A *Salmonella* Montevideo isolate contained an IncHI and an IncN2 plasmid, which both encoded antimicrobial resistance genes. We also identified two novel genomic islands (SGI2 and SGI3), and 42 prophages with mosaic architecture, seven of them harboring known virulence genes. Finally, we identified a novel integrative conjugative element (ICE) encoding a type IVb pilus operon in three non-typhoidal *Salmonella* serovars. Our analyses not only identified a considerable number of mobile elements that have not been previously reported in *Salmonella*, but also found evidence that these elements facilitate transfer of genes that were previously thought to be limited in their distribution among *Salmonella* serovars. The abundance of mobile elements encoding pathogenic properties may facilitate the emergence of strains with novel combinations of pathogenic traits.

## Introduction

*Salmonella* is a widely distributed foodborne pathogen and one of the most common causes of bacterial foodborne illnesses and deaths globally [1]. In the United States, *Salmonella* causes approximately 11% of foodborne illnesses, and is the principal cause of hospitalizations and deaths due to foodborne diseases [1], [2]. The genus *Salmonella* includes two species (*S. enterica* and *S. bongori*) and more than 2,500 different serovars [3]; within *S. enterica*, a total of six subspecies have been reported to date. In addition to species, subspecies and serovar classification, *Salmonella* can also be classified based on their ability to cause disease in different hosts. For example, *Salmonella* has been classified into host-restricted, host adapted, and unrestricted serovars according to the degree of host specificity, or into typhoidal and non-typhoidal serovars, according to clinical presentation of systemic disease in humans [4]–[6]. Typhoidal *Salmonella* serovars (i.e., Typhi, Paratyphi A, B, and C) have been characterized by a unique complement of virulence determinants, including the type IVb pilus operon [7].

Comparisons of the *Salmonella* pan-genome have shown that the sequenced strains share a conserved genomic backbone, and that the vast majority of the genomic variation can be assigned to specific genomic regions in the accessory genome [4]. Mobile elements are part of the accessory genome and have been associated with the emergence of strains with novel pathogenicity phenotypes in a number of foodborne pathogens, including *Salmonella* [8], [9] and *E. coli* [10]. The importance of mobile elements has recently been illustrated by the European *E. coli* O104:H4 outbreak in 2011; this strain appears to have become highly virulent by acquisition of a *stx*2-harboring prophage, as well as a plasmid encoding virulence and resistance determinants [10]. The importance of mobile elements in the evolutionary history of *Salmonella* can be illustrated by the acquisition and rearrangement of different *Salmonella* pathogenicity islands (SPIs) in *S. enterica* and *S. bongori* [11], [12]. In addition to SPIs, which encode genes with experimentally validated virulence functions, genomic islands have been associated with the emergence of endemic strains, for instance, the multidrug resistant phage type *S.* Typhimurium DT104 [13]. Genome analysis of a strain within this phage type has allowed identification of *Salmonella* genomic island 1 (SGI1), which has subsequently been recognized as a globally distributed, integrative mobilizable element containing an array of antimicrobial resistance genes and present in multiple *Salmonella* serovars [8], [13]–[16]. Plasmids have also been shown to play an important role in dispersal and acquisition of virulence and antimicrobial resistance genes in *Salmonella* [17]–[19]. The distribution of plasmids is restricted by their replicons; this forms the basis of classifying plasmids into multiple incompatibility types (Inc types) [20], [21]. Previously reported plasmids in *Salmonella* include IncFIB serovar-specific virulence plasmids and IncA/C conjugative plasmids conferring resistance to multiple antimicrobials [17], [18], [22], [23]. Prophages are common in *Salmonella* and also play important roles in the evolution of this pathogen. For example, *S.* Typhimurium and *S.* Typhi prophages can encode genetic traits that increase pathogenicity (e.g., genes encoding SopE, SodC-1, SspH1, and SseI) or fitness in certain hosts (e.g., genes that lead to O-antigen conversion) [24]–[27].

While different mobile elements (e.g., plasmids, phages, transposons, and mobilizable islands) are clearly important for the evolution of *Salmonella*, including the emergence of strains with novel antimicrobial resistance and pathogenicity-associated phenotypes, our current understanding of mobile element distribution and diversity is still limited. Most descriptions of *Salmonella* mobile elements to date have focused on common or highly virulent serovars (e.g., serovars Typhimurium, Typhi) or strains with multidrug resistance phenotypes. To improve our understanding of mobile element diversity in *Salmonella*, we searched for mobile elements in the genome sequences of 16 different *Salmonella* serovars [5] for which no in-depth mobile element analysis has been performed to date. This study identified new mobile elements in *Salmonella*, including a novel IncI1-IncFIB cointegrated virulence plasmid, two novel genomic islands (SGI2 and SGI3), mosaic prophages carrying virulence genes, as well as an integrative conjugative element encoding the serovar Typhi type IVb pilus operon in three non-typhoidal serovars.

## Results

### Characterization of mobile elements in 16 *Salmonella* serovars

Comprehensive mobile element analysis of the draft genome assemblies of 16 *Salmonella enterica* subsp. *enterica* serovars identified a large number of mobile elements including (i) two antimicrobial resistance plasmids (both found in the same serovar Montevideo strain) and four highly similar virulence plasmids found in four different serovars, (ii) two novel genomic islands (designated SGI2 and SGI3), (iii) three integrative conjugative elements (ICE), and (iv) 35 transposons. In addition, we identified 42 prophages that are each contained in a single sequence contig and thus could be described and characterized with a high level of confidence (Table 1).

**Table 1:** Summary of mobile elements identified in this study.

*Salmonella* serovar	Strain (FSL)	Plasmids	Integrative conjugative elements	Phages^1^	Transposons	Genomic islands	Antimicrobial resistance^2^
Adelaide	A4-669	0	0	2	11	1	Pan-susceptible
Alachua	R6-377	0	0	1	10	1	Pan-susceptible
Baildon	R6-199	0	0	2	8	3	Pan-susceptible
Gaminara	A4-567	0	0	2	12	1	Pan-susceptible
Give	S5-487	0	0	1	11	2	Pan-susceptible
Hvittingfoss	A4-620	0	0	1	8	3	Pan-susceptible
Inverness	R8-3668	1	1	4	9	3	Pan-susceptible
Johannesburg	S5-703	0	0	4	10	2	Pan-susceptible
Minnesota	A4-603	0	0	0	11	2	Pan-susceptible
Mississippi	A4-633	1	0	3	13	3	Pan-susceptible
Montevideo	S5-403	2	0	4	10	2	Strep, Sul, Tet
Rubislaw	A4-653	1	1	4	9	2	Pan-susceptible
Senftenberg	A4-543	0	0	4	11	2	Pan-susceptible
Uganda	R8-3404	0	0	4	7	3	Pan-susceptible
Urbana	R8-2977	1	1	3	11	2	Pan-susceptible
Wandsworth	A4-580	0	0	3	7	1	Pan-susceptible

1 Only phages that were found in a single contig.

2 Pan-susceptible: no resistance detected against any of the antimicrobials tested; Strep: resistance against streptomycin, Sul: resistance against sulfisoxazole, Tet: resistance against tetracycline.

### Identification of antimicrobial resistance gene-carrying plasmids and chromosomally integrated mobile elements that have not been previously reported in *Salmonella*

**Figure 1:**
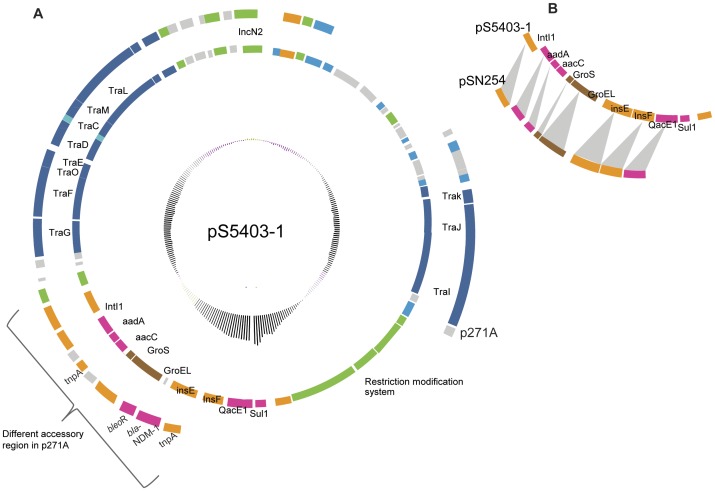
Circular representation of plasmid pS5-403-1 identified in *Salmonella* Montevideo. (A) Circular representation of pS5-403-1. Circles from inside to outside: the inner circle represents the GC%, the second circle is pS5403-1, a 53 kb plasmid that encodes antimicrobial resistance genes, the outer circle represents the shared backbone with p271A plasmid in *E. coli*. The curly bracket represents a cluster of genes in a transposon insertion in p271A that includes the NDM-β-lactamase. (B) Cluster of genes representing a comparison of a class 1 integron in pS5-403-1 homologous to an integron inserted in pSN254 (plasmid in *S.* Newport). Genes were color coded according to function as follows: resistance (pink), plasmid transfer (blue), transposition/IS (orange), replication (green), plasmid stability (turquoise), metabolism (brown), and hypothetical proteins (grey).

Plasmids encoding antimicrobial resistance were only identified in the serovar Montevideo strain FSL S5-403, the only antibiotic-resistant isolate in this set of strains (Table 1). The two plasmids found in this strain–designated pS5-403-1 and pS5-403-2–were predicted *in silico* and subsequently validated by PFGE, PCR, and sequencing. The antimicrobial resistance genes identified on these two plasmids (Fig. 1A and Table 2) were consistent with the resistance phenotype (i.e., resistance to aminoglycosides, tetracycline and sulfonamides). Plasmid pS5-403-1, a 53 kb IncN2 type plasmid, carries the resistance genes *sul1, aacC, aadA, strAB*, and *qacEΔ1* in a class 1 integron (Fig. 1B). This integron shows similarity with a class 1 integron, which was previously reported as a component of pSN254, a plasmid found in multidrug resistant *Salmonella* Newport. Plasmid pS5-403-1 has the same backbone as the *Escherichia coli* plasmid p271A [28] . This backbone has typically been associated with IncN1 plasmids [28]; p271A and pS5-403-1 are the only described IncN2 plasmids with this backbone and, to our knowledge, pS5-403-1 is the first IncN2 plasmid reported for *Salmonella*. Whereas pS5-403-1 has a class 1 integron inserted in the accessory region, p271A has a transposon encoding the New Delhi metallo-β-lactamase, *bla*NDM-1, inserted in this region (Fig. 1A).

**Table 2:** Plasmids predicted and validated in this study.

	Montevideop S5-403-1	Montevideop S5-403-2	Inverness pR8-3668	Rubislaw pA4-653	Mississippi pA4-633	Urbana pR8-2977
Detection method	*de novo*	*de novo* & SIGI-HMM	*de novo*	SIGI-HMM	*de novo*	*de novo*
Validation method	PCR and sequencing	PFGE & long range PCR	PCR and sequencing	PFGE & long range PCR	PCR and sequencing	PCR and sequencing
Size (bp)	53,632	299,644	121,190	152,728	122,532	123,020
GC%	52	48	50	52	51	50
ORFs	66	401	193	218	178	202
Replicon type	IncN2	IncHI1	IncI1/IncFIB	IncI1/IncFIB	IncI1/IncFIB	IncI1/IncFIB
Plasmid stability genes	*ardK, ardB, ccgC,* *cgAI, stbC* *stbA, EcoR124IIMRS*	*parM, parA, parB* *parR, parM, hipA*	*klcA, parB, parA,* *psiB, psiA, hok,* *relE/parE, kfrAs*	*parA, parB, psiB, psiA*	*klcA, parB, parA*, *psiB, psiA, hok, relE/* *parE, kfrAs*	*klcA, parB, parA, psiB, psiA, hok, relE/parE*
Resistance genes	*sul1,qacEΔ, aacC,* *aadA, strAB*	Tn7-like (*pcoE, sil, cus*), *sugE, nodT, ter, tetA*	-	-	-	-
Virulence genes	-	-	*hlyD*, *eal*, adhesin, *pefC*, *ldaA,* *iroN*	*sopE*, adhesin, hemolysin, serine/ threonine phosphatase	*hlyD*, *eal*, adhesin, *pefC, ldaA,* *iroN*	*hlyD*, *eal*, adhesin, *fimCD*

The second plasmid, pS5-403-2 is a large (299 kb) conjugative plasmid of the IncHI replicon type. This plasmid encodes resistance to tetracycline, and predicted resistance to acriflavin, copper, silver, and cadmium. This plasmid shares the backbone region (including regions encoding Tra1 and Tra2 transfer functions and resistance to tellurite, silver, and copper) with previously described IncHI plasmids [29]. Using a guide tree created from an alignment made using the Mauve algorithm (used to cluster plasmids based on their overall identity, see methods) of pS5-403-2 and previously sequenced IncHI plasmids (Table S1), we found that pS5-403-2 is more similar to IncHI plasmids in *E. coli* (pAPCE-O1-R), *Serratia marcescens* (pR478), and *Enterobacter cloacae* (pEC-IMPQ), than to IncHI1 plasmids from *Salmonella* Typhi, Paratyphi A, and Choleraesuis (Fig. S1), indicating that *Salmonella* may have acquired IncHI plasmids from multiple sources.

Interestingly, a Tn7-like transposon inserted in pS5-403-2 carries three heavy metal resistance genes (i.e., *cusR, silE* and *pcoE*) among several other genes (Table 2). A highly similar Tn7-like transposon, which also carries *cusR, silE* and *pcoE*, was identified in *Salmonella* Senftenberg; in this strain this element is chromosomally integrated and inserted at the 3′ end of a gene that encodes a NAD-utilizing dehydrogenase. The transposons found in these two serovars have a similar open reading frame (ORF) content (24 out of 29 ORFs are found in both transposons) and highly similar *tns*ABCD genes (99% nucleotide sequence identity). Two other IncH1 plasmids (i.e., pAPCE-O1-R and pR478) also carry a Tn7-like transposon. Cluster analysis shows that these plasmids are most similar to pS5-403-2 (Fig. S1). Closely related Tn7-like transposons (based on TnsABCD homology) that are chromosomally inserted have been found in *S.* Tennessee str. CDC07-0191, *Escherichia albertii* TW07627, and *Enterobacter cloacae* ATCC 13047 (GenBank accessions NZ_ACBF01000002, ABKX01000002, NC_014121, respectively). Interestingly, in all these organisms Tn7-like transposons are not inserted at the previously described *att*Tn7 site (i.e., downstream of the *glmS* gene) [30], but are inserted at the 3′ end of the gene that encodes a NAD-utilizing dehydrogenase, similar to what we observed for the integration site in the *S.* Senftenberg chromosome. Given that these Tn7 elements are so similar, but found in the exact same location in different genera, it suggests the possibility that they reside in a new attachment site that is recognized by one of the two TnsD proteins encoded in this element.

### IncI1-IncFIB cointegrated plasmids that carry virulence genes in the accessory region are found in serovars that are rarely isolated from animal hosts in the US

**Figure 2:**
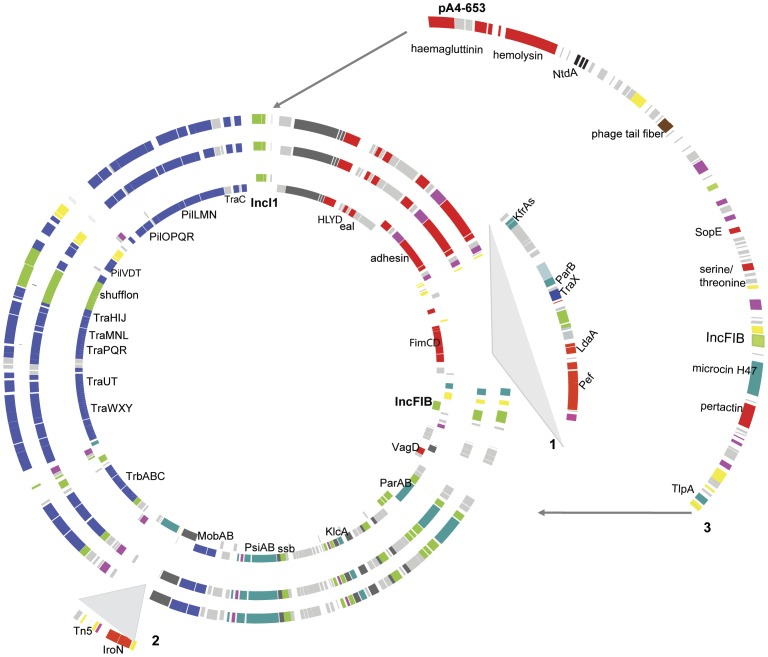
Circular representation of IncI1-IncFIB cointegrated plasmids. Circular representations of IncI1-IncFIB plasmids identified in *Salmonella* serovars Urbana pR8-2977 (inner circle), Inverness pR8-3668 (second circle) and Mississippi pA4-633 (third circle). Plasmids were aligned against the Urbana plasmid. Gene cluster (1) represents insertions in the accessory region in plasmids in serovars Mississippi and Inverness relative to the *S*. Urbana plasmid. Gene cluster (2) represents insertions in plasmids in serovars Mississippi and Inverness relative to the *S*. Urbana plasmid. For plasmid in serovar Rubislaw pA4-653, there is a 90 kb insertion (3) that differentiates this plasmid from other plasmids (outer cluster of genes), and encodes several putative virulence genes, arrows indicate the insertion location for this region in plasmid pA4-653. Genes were color coded according to function as follows: virulence (red), plasmid transfer (blue), transposition/IS (yellow), replication (green), plasmid stability (turquoise), metabolism (purple), phage origin proteins (brown), antibiotic production (black), hypothetical proteins (grey).

In addition to the two resistance plasmids identified in *Salmonella* Montevideo, we identified plasmids with cointegrated IncI1-IncFIB replicons (i.e., “IncI1-IncFIB cointegrated plasmids”) in isolates representing serovars Inverness (plasmid pR8-3668), Mississippi (pA4-633), Rubislaw (pA4-653), and Urbana (pR8-2977). These plasmids were initially predicted by *de novo* assembly as scaffolds without homology to the reference genomes [5] (Table 2 and Fig. 2). We confirmed the presence of these plasmids through *in silico* and experimental approaches, including identification of essential plasmid genes (e.g., genes encoding replication and conjugation functions) through inspection of the annotation of the relevant contigs, PFGE-based estimates of plasmids sizes, and PCR-based validation of a circular molecule (see Materials and Methods for details). These four plasmids have the same backbone previously described for other *Salmonella* and *E. coli* IncI1 plasmids [31]; this backbone includes a type IVa pilus operon as well as genes encoding a type IV secretion system, a shufflon recombinase, an antirestriction protein, plasmid stability proteins, and DNA repair systems (error-prone repair proteins UmuC and UmuD).

While IncI-IncFIIA cointegrated plasmids have previously been described in enterotoxinogenic *E. coli* (ETEC) [20], [31], this is the first description of *Salmonella* plasmids that encode IncI1 as well as the IncFIB replicons. In addition to this unique replicon arrangement, the four IncI1-IncFIB cointegrated plasmids described here are also unique with regard to the virulence gene arrays found in the accessory region of these plasmid (i.e., the region between the replicon and a site-specific recombinase [encoded by LTSEURB_6814 in serovar Urbana]). In the serovars Inverness, Mississippi and Urbana the IncI1-IncFIB cointegrated plasmids contain a region of 20 to 30 kb encoding similar putative virulence proteins such as adhesins, fimbrial proteins, and iron uptake proteins (Table 2). The same region on the IncI1-IncFIB cointegrated plasmid of serovar Rubislaw contains a different set of virulence genes, including *sopE*, as well as genes encoding a serine/threonine phosphatase, a hemolysin, and an adhesin. Moreover, the cointegrated plasmid of serovar Rubislaw also encodes a gene involved in the biosynthesis of antibiotics (i.e., 3,3′-neotrehalosadiamine, *ntdA*). A guide tree created using the Mauve algorithm (used to cluster plasmids based on their overall identity), clustered the four plasmids identified here on a branch that is clearly separated from the branch that contains the previously reported *Salmonella* IncI1 plasmids [20], [31] (Fig. S2). In addition, this comparison showed that four IncI-IncFIB cointegrated plasmids identified here are more similar to the ETEC cointegrated plasmid p557 than to plasmids that carry only the IncI1 replicon.

**Figure 3:**
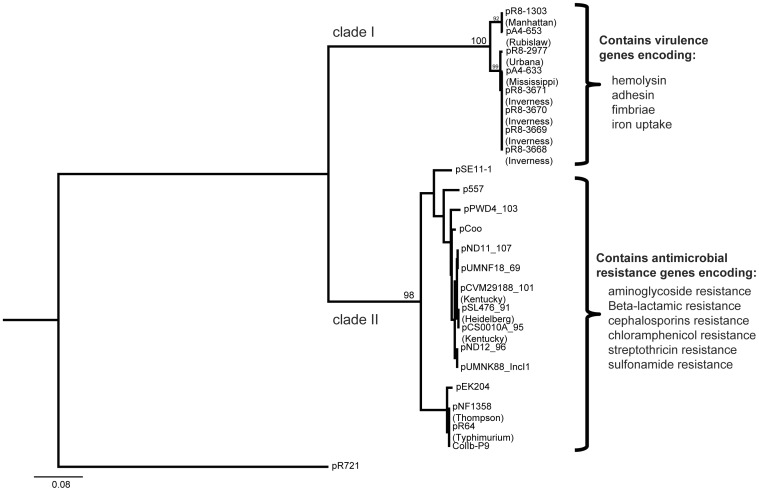
Phylogeny inferred with maximum likelihood of the IncI1 replicon. Maximum likelihood phylogeny conducted with IncI1 sequences for plasmids found in this study and currently available sequences. Analysis was conducted with RAxML. Two clades were observed (clade I and clade II), with clade I containing the *Salmonella* plasmids analyzed here.

PCR assays targeting the IncI1 and IncFIB replicons were used to further screen for the distribution of this IncI1-IncFIB cointegrated virulence plasmid among a broader *Salmonella* subsp. *enterica* population, using a set of 107 isolates representing 84 serovars (Table S2). Nine isolates representing serovars Enteritidis (2 isolates) as well as Rubislaw, Berta, Hindmarsh, Holcomb, Paratyphi C, Wandsworth, and Typhimurium (1 isolate each) were positive for IncFIB replicon, but not for IncI1. Both IncFIB and IncI1 replicons were detected in three serovar Inverness isolates and in one serovar Manhattan isolate (Table 3). The detection of these two replicons could indicate the presence of two plasmids, an IncI1 and an IncFIB, or the presence of an IncI1-IncFIB cointegrated plasmid. For these four isolates, the amplified incI1 fragment (976 bp) was sequenced. The newly sequenced IncI1 sequences, along with the IncI1 sequences for the four IncI1-IncFIB cointegrated plasmids identified by whole genome sequencing and previously reported IncI1sequences (Table S1), were used to construct a IncI1 nucleotide sequence maximum likelihood phylogeny. This phylogeny placed the IncI1 sequences into two well supported clades (98 and 100% bootstrap support) (Fig. 3); (i) a clade (Clade I) containing IncI1 sequences for 8 plasmids (4 Inc1-IncFIB cointegrated plasmids for which the full genome sequence was described here and four plasmids for which only IncI1 was sequenced), and (ii) a clade (Clade II) containing 15 IncI1 sequences, representing 5 *Salmonella* and 10 *E. coli* plasmids which were fully sequenced previously (see Table S1). The eight plasmids of Clade I were obtained from strains classified as serovars Inverness (4 plasmids), Mississippi, Rubislaw, Urbana, and Manhattan. Interestingly, these five serovars are rarely isolated from animal and human hosts in the United States. Based on a CDC report for 2009 [2], [32] these serovars represented only 0.4% of the nonhuman and 1.6% of human isolates obtained over 10 years in the US. The four plasmids in Clade I for which full genome sequences were available contained predominantly putative virulence genes in the accessory region (see Table 2), including *hlyD* (secretion of hemolytic toxins) and *eal* (cellular adhesion) (both found in three of the four plasmids). Two plasmids of the clade II plasmids represented co-integrated plasmids found in *E. coli*. The 5 *Salmonella* serovars represented in this clade are Typhimurium, Thompson, Kentucky, and Heidelberg. Based on a CDC report for 2009 [32] these serovars represented 31% of the 13,006 nonhuman and 22% of human isolates obtained over 10 years in the US. Interestingly, the 15 clade II plasmids did not include any putative virulence genes in their accessory region, but often carried antibiotic resistance genes, including genes conferring resistance to beta lactam antibiotics (e.g., *bla*CTX, *bla*CMY, *bla*TEM [3 plasmids]); aminoglycosides (e.g., *aadA1*, *aadA2*, *strA*, *strB* [3 plasmids]; tetracycline (e.g., *tetA* [3 plasmids]); chloramphenicol (e.g., *cmlA* [2 plasmids]); sulfonamide (e.g., sul2 [1 plasmid]); as well as heavy metals and disinfectants (e.g., *sugE*, *arsR*, *cusR*, *silE*, *qacH* [5 plasmids]). Unfortunately further confirmation of plasmid classification into clade I and II with pMLST, which has been used previously to characterize IncI1 plasmids [33] was not possible as plasmids described here have diverged considerably from IncI1 plasmids. For example, among the five pMLST loci, two loci are absent (i.e., *ardA*, and *sogS*) in these IncI1 plasmids and the other three loci have diverged considerably (e.g., 60–70% for *repI*1).

**Table 3:** Isolates positive for type IVb pili, and/or IncI1-IncFIB replicons.

Isolate FSL no.^1^	Serovars	Type IVb pili	IncFIB	IncI1
R8-3668^b^	Inverness	+	+	+
R8-3669^c^	Inverness	+	+	+
R8-3670^c^	Inverness	+	+	+
R8-3671^c^	Inverness	+	+	+
S5-477^a^	Rubislaw	−	+	−
A4-653^ab^	Rubislaw	+	+	+
R8-2977^b^	Urbana	+	+	+
S5-410^a^	Urbana	−	−	−
S5-661^a^	Urbana	+	−	−
R8-1303^ac^	Manhattan	+	+	+
R6-542	Manhattan	+	−	−
R8-1550^a^	Manhattan	−	−	−
R6-305	Paratyphi C	+	+	−
A4-633^b^	Mississippi	−	+	+

1 Isolates used for invasion assay are marked with ^a^, isolates with completed whole genome sequences are marked with ^b^, isolates were IncFIB and IncI1 replicons were identified by PCR and sequencing are marked with ^c^ (which could indicate the presence of both an IncI1 and an IncFIB plasmids or the presence of a cointegrated plasmid).

### Identification of novel genomic islands and transposons encoding genes that may contribute to host specificity and *Salmonella* transmission

We used SIGI-HMM [34] and comparative genomic analyses with the Mauve algorithm and RAST [35]–[37] to predict and identify putative genomic islands among the 16 genomes analyzed here. As expected, a number of putative *Salmonella* genomic islands were identified using this approach. We defined genomic islands as chromosomal regions more than 15 kb in length, that represent variable gene content across *Salmonella* strains and differ in codon usage compared to the rest of the genome, which is a definition similar to Juhas et al. [38]. To focus on novel genomic islands that may be relevant for emergence of strains with unique pathogenicity characteristics, we did not further analyze regions representing previously reported *Salmonella* pathogenicity islands (SPIs). The remaining nine putative genomic islands were designated as *Salmonella* genomic islands (SGI) 2 to 10; SGI1 was previously assigned to a genomic island that encodes antibiotic resistance genes found in *S.* Typhimurium DT104. One putative genomic island (SGI4) was found in most *Salmonella* genomes reported so far, while six putative genomic islands (SGI6 to 10) did not contain apparent features relevant to host specificity or transmission (see Table S3 for more details on these islands). However, we identified two novel genomic islands in these 16 genomes (SGI2 and SGI3) that contained genes that may be linked to unique host specificity and transmission characteristics and in particular an ability to interact with plant hosts. These two regions are discussed in more detail below.

SGI2 was predicted in nine genomes representing *Salmonella* serovars Montevideo, Johannesburg, Urbana, Baildon, Uganda, Minnesota, Mississippi, Give, and Hvittingfoss. The insertion site for SGI2 was identified as tRNA-leu (CAA), except in serovar Hvittingfoss (Table 4). SGI2 always encodes one or two toxin-antitoxin modules and different repertoires of restriction-modification systems, suggesting similar functionality despite a difference in gene repertoire. SGI2 shows high sequence similarity only in three genomes (serovars Montevideo, Urbana, and Johannesburg). However, distinct variants with conserved regions of SGI2 were found in the other six genomes, and were designated as SGI2.1 to SGI2.6 (Fig. S3). Four of these variants encode genes that may facilitate propagation in animal and plant hosts, including SGI2.3 (Give), SGI2.4 (Baildon), SGI2.5 (Uganda), and SGI2.6 (Hvittingfoss) (Table 4) [39]–[41]. SGI2.3 and SGI2.5 encode different guanine-binding proteins; SGI2.4 carries a gene encoding a pectin lyase fold protein, which may facilitate interaction with plant hosts, and SGI2.6 includes an insertion of a toll-interleukin receptor (see discussion for specific interpretations).

**Table 4:** Characteristics of SGI2 variants.

SGI	Serovars	Size(kb)	Inser-tion tRNA	RM type	Toxin- antitoxin	Virulence proteins	Resistance proteins	DNA repair
SGI2	Montevideo	19	Leu-CAA	I^1^	YpjF-YfjZ	-	-	-
	Johannesburg							
	Urbana							
SGI2.1	Minnesota	23	Leu-CAA	II^2^	CcdAB	-	-	-
SGI2.2	Mississippi	25	Leu-CAA	II	YpjF-YfjZ & CcdAB	-	-	RadC
SGI2.3	Give	31	Leu-CAA	III	CcdAB	Guanine nucleotide binding protein	-	-
SGI2.4	Baildon	35	Leu-CAA	I	YpjF-YfjZ & CcdAB	Pectin lyase, antigen 43	-	RadC
SGI2.5	Uganda	42	Leu-CAA	II^2^	YpjF-YfjZ	Guanine nucleotide binding protein	Bleomycin resistance	RadC
SGI2.6	Hvittingfoss	17	Sec-TCA	I^1^	YpjF-YfjZ	Toll-interleukin receptor	-	RadC

1 SGI2 and SGI2.6 share the same type I restriction modification system.

2 SGI2.1 and SGI2.5 share the same type II restriction modification system.

RM: restriction modification system.

SGI3 is a novel 31 kb genomic island that was only identified in the serovar Mississippi genome. SGI3 is inserted at the 3′ end of the GDP-mannose pyrophosphorylase gene, and resembles a region in the chromosome of *Yersinia intermedia* ATCC 29909 (GenBank accession NZ_AALF00000000). SGI3 encodes an integrase, transposases, OpgC (succinyl modification of osmoregulated periplasmic glucan), a cellulose synthesis protein, beta-galactosidase, and several proteins involved in stress response and regulation (i.e., sensory box histidine kinase/response regulator, an anti-sigma factor antagonist, and a serine phosphatase regulator) (see discussion for specific interpretations).

In addition to the genomic islands described above, we also identified 35 putative transposons (<1 kb to >15 kb), which encode a range of proteins that may facilitate transmission by favoring their maintenance, including restriction modification systems, efflux pumps, O-antigen conversion, and disinfectant resistance (Table S4). One of these transposons, a 10 kb Tn-31-like transposon, was present in all 16 genomes. This transposon encodes a potassium efflux system and also contains an operon encoding acriflavin resistance. In 11 genomes (serovars Baildon, Gaminara, Give, Inverness, Johannesburg, Mississippi, Montevideo, Senftenberg, Uganda, Urbana, and Wandsworth), we identified a transposon insertion downstream of SPI-1. This transposon encodes genes involved in transposition and a serine/threonine specific phosphatase-1.

### Identification of prophages with highly mosaic genome architecture in selected *Salmonella* serovars

Lysogenic phages and/or remnants of phages were initially detected by Prophinder [42] in all the 16 *Salmonella* genomes, and subsequently confirmed *in silico* by manual annotation and comparative analysis. Only phages that were found in a single contig and with a size of at least 20 kb were further analyzed. A total of 42 putative phages were identified in the 16 genomes; these phages were classified into 12 groups; 11 groups represent phages that are similar to previously reported phages (e.g., PSP3-like, P22-like, Gifsy-1-like), while 1 group comprises 6 phages that do not show similarity with any previously reported *Salmonella* phages (Table S5).

**Figure 4:**
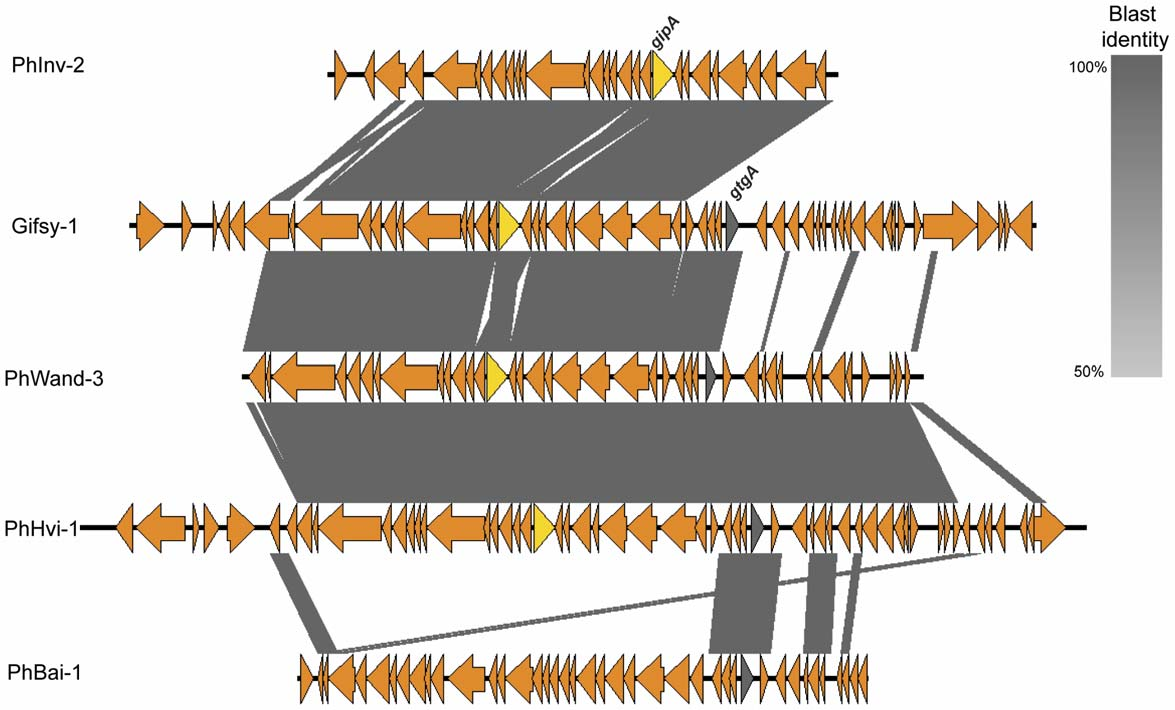
Representation of prophages carrying virulence genes. Blast comparison of Gifsy-1-like prophages detected in the 16 genomes. Prophage order from top to bottom is PhInv-2 (*S.* Inverness), Gifsy-1 (*S.* Typhimurium LT2), PhWands-3 (*S.* Wandsworth), PhHvi-1 (*S.* Hvittingfoss), and PhBai-1 (*S.* Baildon). Arrows in orange represent coding regions, and grey shaded regions represent regions with homology. The two virulence genes (*gipA* and *gtgA*) are labeled in yellow and grey, respectively.

Analysis of “morons” (i.e., genes not required for the phage infective cycle) [43], [44] among the 42 phages, identified genes encoding DNA-methylases in 15/42 phages (including PSP3-like, HP2-like, Fels2-like, PhiCTX-like, and HK97-like phages) as well as genes with potential functions in fitness and virulence. For instance, O-antigen conversion genes were identified in five phages (see Table S5). While all four Gifsy-1-like phages identified here (i.e., PhBail-1 in *S.* Baildon, PhWands-3 in *S.* Wandsworth, PhHvi-1 in *S.* Hvittingfoss, and PhInv-2 in *S.* Inverness) carried putative virulence genes, Gifsy-1 does not appear to be fully conserved in these four genomes. The two virulence genes previously reported in Gifsy-1-*gtgA* and *gipA*, which facilitate *Salmonella* growth or survival in Peyer's patches and enterophatogenesis [45]–[47]-, are both present in PhWand3 and PhHvi1, while only *gtgA* and only *gipA* are present in PhBai-1 and PhInv-2, respectively (Fig. 4).

**Figure 5:**
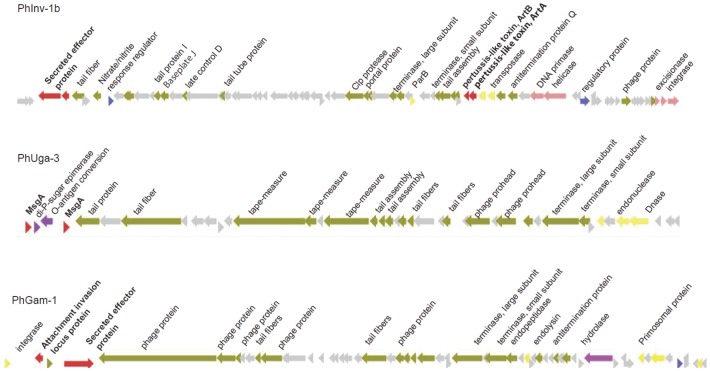
Linear representations of three phage genomes identified to have virulence genes. At the top is phage PhInv-1b in *S.* Inverness, the middle is phage PhUga-3 in *S.* Uganda and in the bottom is PhGam-1 in *S.* Gaminara. Genes were color coded according to function as follows: virulence (red), replication (yellow), phage structural genes (green), metabolism (purple), and hypothetical protein (grey).

In addition, we identified putative virulence genes in three different phages in the genomes of serovars Inverness, Uganda and Gaminara. In *S.* Inverness, we identified a 48 kb phage (PhInv-1b) that encodes a secreted effector protein and the pertussis-like toxin ArtAB. In *S.* Uganda, we identified a 29 kb phage resembling HK97 (PhUga-3) that encodes two copies of virulence protein *msgA* and an O-antigen conversion protein. Finally, we identified a 52 kb phage in *S.* Gaminara (PhGam-1), which resembles a Stx-2 phage. While this prophage does not encode Stx-2, it does encode a secreted effector protein and an attachment invasion protein (Fig. 5).

**Figure 6:**
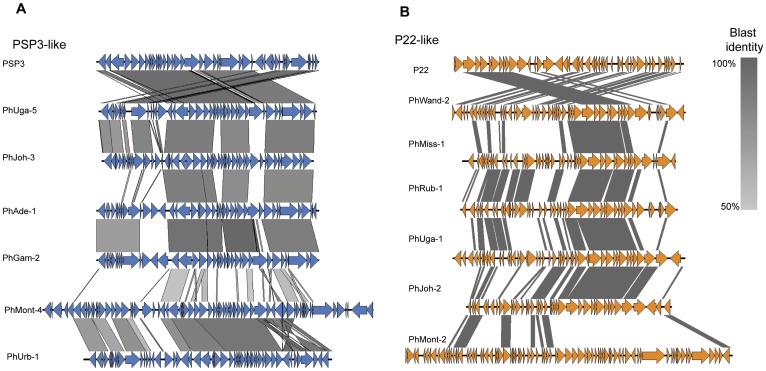
Comparison, made using the Blast algorithm, of PSP3-like and P22-like phages. (A) Comparison of six phages that resemble PSP3; phages were identified in serovars Uganda, Johannesburg, Adelaide, Gaminara, Montevideo, and Urbana. Coding regions are represented as blue arrows and regions of homology are shaded in grey. (B) Comparison of six phages that resemble P22, identified in serovars Wandsworth, Mississippi, Rubislaw, Uganda, Johannesburg, and Montevideo. Coding regions are represented as orange arrows and regions of homology are shaded in grey.

Comparison of phages belonging to the same group indicated a highly mosaic composition of the phage genomes. For instance, a comparison of six phages that resemble Enterobacteria phage PSP3 shows that phages in the genomes of serovars Uganda (PhUga-5), Johannesburg (PhJoh-3), Adelaide (PhAde-1), and Gaminara (PhGam-2) are conserved and more similar to phage PSP3, while related phages in Urbana (PhUrb-1) and Montevideo (PhMont-3) are more distinct from these phages and PSP3 (Fig. 6A). Both PhMont-3 and PhUrb-1 have several gene duplications (i.e., eight in PhMont-3 and six in PhUrb-1), they have nine genes in common that are absent in the rest of phages in this group, as well as 8 and 4 new unique genes, respectively. Similar to the PSP3-like phages, a mosaic architecture was also identified in the six phages that resemble *Salmonella* phage P22 (Fig. 6B).

### ICES1, a novel *Salmonella* integrative conjugative element, encodes a *Salmonella* Typhi virulence factor, the type IVb pilus operon

**Figure 7:**
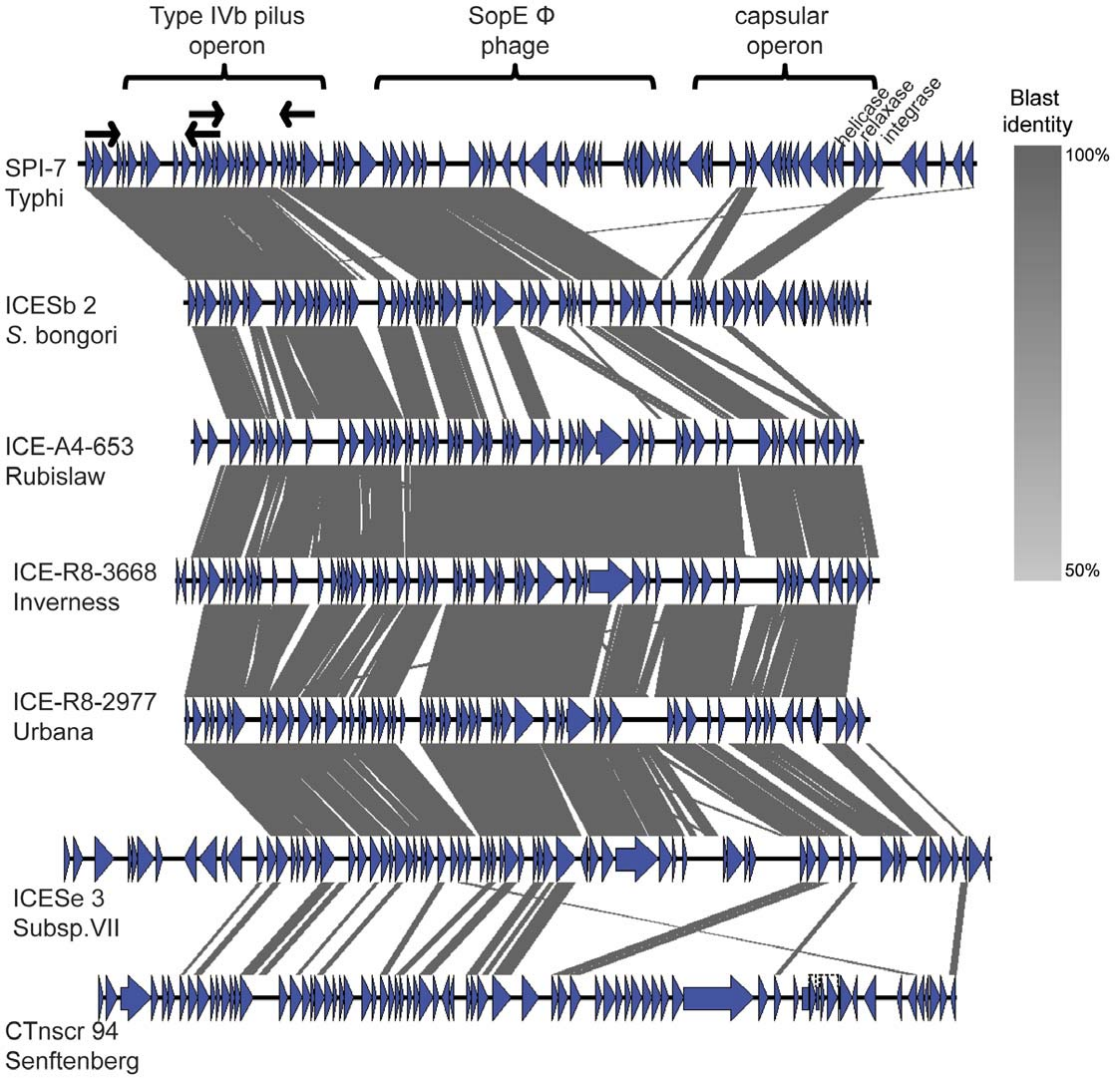
Comparison, made using the Blast algorithm, of SPI-7 in *S.* Typhi, ICES1 identified in this study, and previously sequenced ICE. At the top is SPI-7 of *S.* Typhi with its three main regions (i.e., type IVb pilus operon, SopEΦ phage and capsular operon). Black arrows indicate the position of the primers used to validate the presence of the type IVb pilus operon in ICES1 in *S.* Rubislaw, *S.* Inverness and *S.* Urbana. Blue arrows are the coding regions and grey shaded are regions that present homology.

We identified a novel mobile element in the genomes of the three serovar Inverness, Urbana and Rubislaw strains (Fig. 7). This novel element is a putative integrative conjugative element (which we designated ICES1) inserted adjacent to the tRNA-CAA gene in these three genomes. ICES1 is flanked on one side by an integrase, and encodes phage genes (e.g., activator of prophage gene expression IbrB) as well as genes involved in transfer of the ICE (e.g., genes encoding an ICE relaxase and a conjugation system). ICES1 carries a type IVb pilus operon that resembles the *Salmonella* Typhi SPI-7, island which also has the SopEΦ prophage and the capsular operon [48]. The full type IVb pilus operon is present in the *Salmonella* Inverness, Urbana and Rubislaw ICES1, however the prophage is incomplete, and the capsular operon (found in *S.* Typhi) appears to be absent. The presence and location of the full type IVb pilus operon in these three strains was also experimentally confirmed using a long-range PCR mapping strategy. Recently, an ICE with similar characteristics was reported in *Salmonella bongori* as well as *S. enterica* serovars Senftenberg, Hadar, and *S. enterica* subspecies VII [49] (Table S1); all three of these ICEs also encode the type IVb pilus operon, which is less conserved in *S.* Senftenberg. Phylogenetic analysis based on the sequences of the ICES1-encoded *pilQ* found in this study and publicly available *pilQ* sequences revealed three *pilQ* clades with low sequence divergence within clades, including (i) a clade consisting of serovar Typhi, Paratyphi C, Dublin, and *S. bongori* (ii) a divergent clade composed of serovars Inverness, Urbana, Rubislaw, and subspecies VII, and (iii) a separate clade representing serovar Senftenberg (Fig. S4). This phylogenetic pattern suggests multiple horizontal gene transfer events of the type IVb pilus operon; in particular the serovar Senftenberg type IVb pilus operon appears to have a distinct origin, while the type IVb pilus operons in serovars Typhi, Paratyphi C, Dublin, and *S. bongori* may represent another origin.

To further probe the distribution of the type IVb pilus operon among a broader *S. enterica* population, a set of 107 isolates representing 84 serovars was screened, using PCR, for the presence of the most conserved genes in the type IVb pilus operon (i.e., *pilQ*, *pilV*, and *rci*). Six of the 107 isolates were positive for all three genes including 3/3 serovar Inverness and 2/5 Manhattan isolates as well 1/2 Urbana isolates tested. One Paratyphi C isolate tested positive for *pilQ*, but negative for *pilV* and *rci* (Table 3), which are less conserved than *pilQ*.

Based on these data, we investigated whether isolates encoding the type IVb pili may show a particular ability to invade human intestinal epithelial Caco-2 cells. Caco-2 invasion assays were performed with (i) one Manhattan isolate, one Urbana isolate, and one Rubislaw isolate, that all contained the type IVb pilus operon, and (ii) one Manhattan isolate, one Urbana isolate, and one Rubislaw isolate that did not contain the type IVb pilus operon (as supported by the absence of *pilQ*, *pilV*, and *rci;* Table 3). No difference (p = 0.347) in invasion efficiency was observed between isolates with or without the type IVb pilus operon.

Remarkably, all four isolates of *Salmonella* Inverness tested in this study that were positive for the type IVb pilus operon (as supported by the presence of *pilQ*, *pilV*, and *rci*) were also positive for the IncI1 and IncFIB replicons, suggesting presence of two plasmids (one IncI1 and one IncFIB) or of a IncI1-IncFIB cointegrated plasmid (see above), further validation is needed to confirm if these replicons are found on two or one plasmid. While this could indicate that these four serovar Inverness isolates represent the same clonal strain, XbaI PFGE analysis revealed four different PFGE patterns for these isolates (Fig. S5), suggesting that they may not be closely related. The presence of both the type IVb pilus operon and IncI1-IncFIB replicons may therefore be conserved in the genome of serovar Inverness.

## Discussion

*Salmonella* strains are subject to frequent integration of new mobile elements. These integration events may give rise to strains with novel pathogenic phenotypes and are often associated with the emergence of new endemic or epidemic strains. Genome scale prediction of mobile elements conducted in this study using 16 different *Salmonella* serovars [5] for which no in-depth mobile element analyses have been available to date, considerably expands our understanding of the diversity of mobile elements found in this important foodborne and animal pathogen. Key findings from this study include (i) a new type of co-integrated *Salmonella* plasmid, which is found among multiple serovars, that contains accessory genes with putative virulence functions, (ii) previously unidentified genomic islands and prophages that encode functions that may facilitate *Salmonella* propagation among animal and plant hosts, and (iii) an ICE encoding the type IVb pilus operon, which could facilitate transfer of this *Salmonella* Typhi virulence factor to non-typhoidal *Salmonella* serovars.

### A new type of co-integrated *Salmonella* plasmid, which is found among multiple serovars, contains accessory genes with putative virulence functions

In this study we identified a type of IncI1-IncFIB cointegrated plasmid that has not previously been reported in *Salmonella*. A key unique feature of this plasmid is that it contains both the IncI1 and the IncFIB replicon, but contains the IncI1 backbone; this plasmid thus represents a IncI1-IncFIB cointegrated plasmid. IncI1 and IncFIB plasmids have previously been described in *Salmonella* [17], [50]. Other cointegrated plasmids previously reported in *Salmonella* include IncFIIA-IncFIB in *Salmonella* Kentucky (pCVM29188_146), as well as the *Salmonella* virulence plasmid in serovars Typhimurium, Enteritidis and Choleraesuis [17], [50]. The presence of multiple replicon types was recently reported for F plasmids in *E. coli* [51], indicating that the presence of multiple replicons could be a characteristic of F plasmids. The IncI1-IncFIB cointegrated plasmid described here has a novel array of virulence genes in the accessory region. Prior to our description of this plasmid, the accessory region of IncI1-plasmids, which have the same backbone as the IncI1-IncFIB cointegrated plasmid described here, was found mostly to contain antimicrobial resistance genes and specifically genes that encode resistance to cephalosporins [20].

Interestingly, we also found initial evidence for differential accessory gene “content” on related plasmids with the IncI1 replicon. IncI1-IncFIB plasmids that were obtained from *Salmonella* serovars that are rarely isolated from food-producing animals in the US (i.e., serovars Inverness, Rubislaw, Mississippi, and Urbana) [2], [32] all harbored virulence genes in the accessory region of the plasmid. For example, three of these plasmids harbor *hlyD*, encoding a protein essential for secretion of hemolytic toxins in *E. coli* [52], and *eal*, which encodes a protein involved in cellular adhesion [53]. On the other hand, plasmids with an IncI1 replicon that grouped into a separate Clade (clade II, Fig. 3) typically have antimicrobial resistance genes integrated in the accessory region of these IncI1 plasmids [31], [50], [54], [55]. The *Salmonella* isolates that carried these antimicrobial resistance plasmids represent serovars commonly isolated from food-producing animals, including serovars Typhimurium, Kentucky, Heidelberg and Thompson [2]. These observations suggest that additional studies on accessory genes of plasmids found in serovars that are predominant in different host types will provide further insights in the evolution of antibiotic resistance and virulence associated characteristics in *Salmonella*.

In addition to IncI1-IncFIB cointegrated plasmids, we also identified two additional plasmids in *S*. Montevideo FSL S5-403. This is the only strain that showed phenotypic antibiotic resistance in this study. This *Salmonella* Montevideo strain contained an IncHI plasmid that is more similar to *E. coli* and *E. cloacae* plasmids, than to IncHI plasmids previously identified in *Salmonella* serovars Typhi and Paratyphi A [56]–[58]. This *Salmonella* Montevideo strain also contained an IncN2 plasmid (pS5-403-1). In addition to this description, IncN plasmids carrying antimicrobial resistance (quinolone) were previously reported in four *Salmonella* serovars (i.e., Bredeney, Typhimurium, Saintpaul, and Kentucky) [59]. Importantly, the IncN2 plasmid identified here shares its backbone with a plasmid that was previously identified in *E. coli* as carrying the *bla*NDM-1 extended spectrum beta-lactamase, which confers resistance to multiple antimicrobials [28]. *bla*NDM-1 was also recently identified in a single *Salmonella* isolate from the United States using PCR [28], [60], but the genomic location of this gene was not identified. Identification of an IncN2 plasmid with an identical backbone to the plasmid that was previously found to carry *bla*NDM-1, suggests that *Salmonella* has the potential to acquire the *E. coli* IncN2 plasmid carrying the *bla*NDM-1.

### Previously unidentified genomic islands and prophages may encode functions that facilitate *Salmonella* propagation among animal and plant hosts

In this study we identified two novel genomic islands; one of which (SGI2) has evolved six different variants. SGI2 is conserved in only three of the genomes. These three genomes represent closely related serovars (i.e., Montevideo, Johannesburg, and Urbana) classified into the clade B group proposed by den Bakker et al. [5]. Interestingly, different SGI2 variants harbor genes encoding proteins with similar functions, including restriction modification systems [61], [62], toxin-antitoxin systems [61], [63]–[65], and genes partially involved in host specificity and virulence, even though the specific genes encoding a given function can differ considerably between SGI2 variants. For example, different SGI2 variants encode different restriction modification (RM) systems, including two different type I RMs, two different type II RMs, and one type III RM, consistent with previous reports that RM system substitution is common in bacterial genomes and with a report of a cassette-like variations of restriction enzyme genes in the same genome region in several *E. coli* strains [61], [62], [66]. SGI2 is inserted at the tRNA-Leu (CAA) in eight of the nine genomes, suggesting that this region is an insertion “hot spot” for genomic islands that differ in gene content, but that are very similar in the functions they encode. Remarkably, tRNA-Leu (CAA) has been previously defined as a highly variable hot spot for gene acquisition in *Salmonella enterica*, including restriction modification systems [67].

Interestingly, SGI2 also appears to encode distinct propagation associated functions in different genomes. For instance, SGI2 variants in serovars Give and Uganda encode a guanine-binding protein, while the serovar Hvittinfgoss SGI2 encodes a toll-interleukin receptor. These proteins have been shown to affect cellular functions involved in host intracellular signaling and in the mammalian immune response [40], [68], [69], suggesting a possible role in infection of mammalian hosts. The serovar Baildon SGI2 encodes a pectin lyase, an enzyme that degrades the plant cell wall and thus may facilitate bacterial invasion of plant cells [39]. Interestingly, the serovar Mississippi SGI3 also appears to encode functions that may facilitate interaction with plant hosts, including a cellulose synthesis protein, which was shown to be required for adherence and aggregation to plant roots in *Agrobacterium* [70] and OpgC (osmoregulated periplasmic glucan), which is also associated with host-pathogen interaction in plant pathogens [71], [72]. While our findings suggest that SGI2 and SGI3 encode functions that may facilitate interactions with plant and animal hosts, phenotypic characterization and infection studies in different hosts are needed to test this hypothesis and to better understand the role of mobile elements in transmission of *Salmonella* among animal and plant hosts.

Previous reports of *Salmonella* phage-borne virulence genes have focused on *Salmonella* Typhimurium, Typhi and Paratyphi [24], [27], [73] and have revealed important roles of prophages in the pathogenicity of these serovars. For example, Gifsy-2 prophage has two major virulence determinants (i.e., *sodC1* and *sseI/gtgE/srfH*), that increase the capability of *S.* Typhimurium to produce systemic infection in mice [46], [74]. In this study, we found a number of phage morons that encode virulence factors, which have previously been thought to be limited to a few serovars. For example, we identified, in the serovar Inverness genome, a 48 kb phage that encodes a pertussis-like toxin, ArtAB; a phage with this putative virulence gene has previously only been reported in *S.* Typhimurium DT104 [73]. Our comparative analysis of the chromosomally inserted phage genomes reported here along with previously reported *Salmonella* prophages also found Gifsy and λ-like phages with putative virulence genes (e.g., *sopE*, *sodC1*, *gtgA*) in a number of serovars. We also identified six phages of the P22 family, including two that encode for O-antigen conversion genes, consistent with previous studies that found O-antigen conversion genes in P22 phage genomes [75]–[77].

### An ICE encoding the type IVb pilus operon could facilitates transfer of this *Salmonella* Typhi virulence factor to non-typhoidal *Salmonella* serovars

In this study we described a unique mobile element in *Salmonella* that harbors *S.* Typhi virulence genes. This mobile element, which we designated ICES1, encodes the type IVb pilus operon, an important virulence factor that facilitates *S.* Typhi invasion of human intestinal cells [78], [79]. While we identified this mobile element in the genomes of isolates representing serovars Inverness, Rubislaw, and Urbana, another recent study [49] also identified a very similar mobile element, which also encodes the type IVb pilus operon, in *S. bongori* as well as in *S. enterica* serovars Senftenberg and Hadar and a subspecies VII isolate. While a unique pathogenicity island repertoire (i.e., presence of SPI-18, SPI-7 and the *cdtB* islet) has typically been considered to be responsible for the unique clinical symptoms associated with *S.* Typhi infection [4], [5], [80]–[82], our study, along with other recent studies [5], [49], provides increasing support for the notion that *S.* Typhi virulence factors are more widely distributed among non-typhoidal *Salmonella* serovars than previously assumed and are often present on mobile elements, which facilitate dispersal.

While two types of type IV pili (i.e., IVa, and IVb) have been described in different Gram-negative and Gram-positive bacteria (e.g., *Neisseria gonorrhoeae, Pseudomonas aeruginosa*, *Mycobacterium bovis*, and *Vibrio cholerae*) [83], type IVb pili have only been reported in human pathogens [83]. Type IVa pili, on the other hand, have been detected in a wider range of bacteria. Numerous virulence-related functions are associated with type IV pili, including surface motility, bacterial aggregation, biofilm production, adhesion, invasion, and immune evasion [83]. In *S.* Typhi, the type IVb pilus operon is located in SPI-7, an island containing several independent mobile regions (i.e., the pilus operon, the SopEΦ phage, and the capsular operon) [4], [84]. Our finding of ICES1, along with the recent description of a very similar element in additional *Salmonella* serovars [49], indicates putative horizontal gene transfer of the type IVb pilus operon across diverse *Salmonella* strains. Dispersal of the type IVb pilus operon was also supported by phylogenetic analysis of its nucleotide sequences, which showed lower sequence divergence in the type IVb pilus operon among serovars Inverness, Rubislaw, and Urbana than expected given their phylogenetic relationships previously described [5]. This indicates likely recent transfer of the type IVb operon between these strains [49]. Horizontal transfer of this mobile element containing the IVb pilus operon is also supported by the recent report showing presence of this element in additional *Salmonella* serovars, and a demonstration of transfer by conjugation from the host strain into *S.* Typhimurium [49]. If one key trait making typhoidal strains pathogenic is also found in non-typhoidal strains, this suggests that a combination of traits makes typhoidal strains so dangerous [11]. Given that many of these traits are on mobile elements, it makes the emergence of new pathogenic strains likely.

While a diversity of virulence associated genes have been found in different *Salmonella* serovars [4], [5], [11], [12], our understanding of the association of virulence and host specificity associated phenotypes with different gene repertories in *Salmonella* is still limited. In this study we found the presence of IncI1-IncFIB cointegrated plasmids to be associated with the genomic presence of the type IVb pilus operon and ICES1. Remarkably, the IncI1-IncFIB cointegrated plasmid encodes a type IVa pilus operon, indicating that at least some strains classified into serovars Inverness, Rubislaw, and Urbana encode both type IVa and IVb pili; for serovar Inverness it appears that both of these pili are found in a several strains within this serovar, as supported by the PCR-based population screen performed here. To our knowledge this is the first report of presence of both of these pili in *Salmonella* strains. Future experiments focusing on the expression and the potential roles in virulence and transmission of these two pili in *Salmonella* strains, that encode both pili, are warranted to probe the functional importance of these genetic elements.

## Materials and Methods

### Isolates

We analyzed 16 *Salmonella* serovars that were previously sequenced with the SOLiD™ next generation sequencing technology by our group [5]. These isolates represent the serovars Montevideo, Inverness, Rubislaw, Give, Mississippi, Urbana, Uganda, Senftenberg, Gaminara, Baildon, Minnesota, Hvittingfoss, Adelaide, Alachua, Wandsworth, and Johannesburg. For details about sequencing workflow, isolate sources, and accession numbers see den Bakker et al. [5].

Antimicrobial susceptibility was determined according to the National Antimicrobial Resistance Monitoring System (NARMS) protocol, at the New York State Animal Health Diagnostic Center (NYSAHDC). Minimal inhibitory concentrations (MIC) were determined using the Sensititre system (TREK Diagnostic Systems, Cleveland, OH), for the following 15 antimicrobials: amikacin, ampicillin, amoxicillin clavulanate, cefoxitin, ceftiofur, ceftriaxone, chloramphenicol, ciprofloxacin, gentamicin, kanamycin, nalidixic acid, streptomycin, sulfisoxazole, tetracycline and trimethoprim-sulfonamide. MIC values were interpreted according to the Clinical and Laboratory Standards Institute (CLSI).

Previously described transposable element nomenclature was used to classify chromosomally inserted mobile elements [38], [85], [86]. Briefly, transposons were classified as either composite or unit transposons [86], integrative conjugative elements (ICE) were defined as chromosomally inserted transposons that carry genes for insertion, excision and conjugative transfer [85], [86], and genomic islands were defined as relatively large regions of DNA (>15 kb), that presented variable gene content across *Salmonella* strains, and that were recognized by having different nucleotide codon usage than the rest of the genome [38].

### Prediction of mobile elements

To predict mobile elements, we prepared a pseudogenome (contigs after scaffolding were connected with a pseudomarker “NNNCACACACTTAATTAATTAAGTGTGTGNNN” that was added to identify the different scaffolds). These pseudogenomes were used for mobile element prediction. We used SIGI-HMM, a program that identifies regions that differ in codon usage compared to the rest of the genome [34], and Prophinder, a web server that compares query sequences against a phage database [42]. This methodology was combined with a search for mobile element-related key words from the RAST annotations (e.g., integrase, transposase and phage). Most plasmids were found as large scaffolds that could not be aligned to the reference genome. These large scaffolds were considered putative plasmids and were further analyzed by comparative analysis. Briefly, RAST annotations were manually examined for essential plasmid backbone genes (e.g., genes involved in plasmid partitioning and replication). All the predicted mobile elements were analyzed by comparative analysis using a Mauve algorithm [35], [36], and with the comparative analysis tools of RAST [37]. Sequence data from selected plasmids, prophages, genomic islands and transposons were extracted from pseudogenomes. Because these are draft genomes, there is not an easy option to deposit these predicted elements in GenBank; however, all contigs are available in GenBank (see den Bakker et al.) [5]. For the predicted elements (plasmids, genomic islands, and prophages), sequences were extracted from the pseudogenome, and genbank files with the sequences and annotations of selected mobile elements are available at https://confluence.cornell.edu/display/FOODSAFETY/CornellFoodSafetyLaboratoryMicrobialGenomeData.

### Comparative and phylogenetic analysis of predicted plasmids

IncI1-IncFIB cointegrated plasmids were compared with previously sequenced plasmids of the IncI1replicon in *Salmonella* and *E. coli* (Table S1). Plasmids were aligned with the Progressive Mauve algorithm, and the guide tree was used to as an indicator of overall sequence similarity of these plasmids. Briefly, Mauve calculates a guide tree, which is a neighbor joining tree computed based on an estimate of the shared sequences among each pair of input genomes. In addition, a maximum likelihood phylogeny was inferred based on the replicon nucleotide sequences, using RAxML 7.03 [87]. The nucleotide substitution model was a general time reversible model with a gamma parameter for rate heterogeneity. For the two plasmids predicted in *Salmonella* Montevideo, we compared each plasmid individually with similar publicly available plasmids using Mauve and the comparative tools in RAST.

### Prophage comparative analysis

Prophages of at least 20 kb predicted by prophinder were extracted from the original contigs, but only if they were found in a single contig. Sequences were annotated in RAST and comparative analyses were performed as described above. Figures were prepared with Easyfig [88].

### PCR confirmation of predicted mobile elements

Predicted plasmids were validated by PCR. Regions of approximately 500 bp from the contig's end and start on predicted plasmids were used as template for primer design. A PCR with these newly developed primers was used to validate the circular conformation of plasmids. Primer sets and PCR conditions are provided in Table S6. To confirm that the amplicon obtained correspond to plasmids, PCR products were purified (Exonuclease I and shrimp alkaline phosphatase [USB, Cleveland, OH] and sequenced (Applied Biosystems Automated 3730 DNA Analyzer at the Cornell University Life Sciences Core Laboratories Center). Sequences were assembled with SeqMan (DNAStar Inc., Madison, WI), and overlap with the end and start of the plasmid contig was examined. Two plasmids were not validated with this approach (i.e., pA4-653 in serovar Rubislaw, and pS5-403-2 in serovar Montevideo). For these two plasmids we used long-range PCR, and estimated the size of the plasmid with PFGE as described below. Takara LA Taq (Takara BIO Inc., Shiga, Japan) was used for long-range PCR, according to manufacturer recommendations.

### Pulsed field gel electrophoresis estimation of plasmid sizes

Plasmids on serovars Montevideo (pS5-403-2) and Rubislaw (pA4-653) were not validated by the traditional PCR approach. To obtain an approximation on the size of these plasmids, we conducted PFGE. Briefly, plasmids were extracted using Plasmid Midi Kit (Qiagen Inc., Valencia, CA). After isolation, two plugs were prepared per isolate by mixing 80 ng/µl and 160 ng/µl of plasmid DNA with melted 1.0% of SeaKem Gold (SKG) agarose (Lonza Walkersville Inc., Walkersville, MD) in TE (pH 8.0). In silico restriction of the predicted plasmids was performed with NEBcutter V2.0, and enzymes that cut one or two sites in the predicted plasmids were used. Specifically, *Not1* and *Fse1* were used to digest pS5-403-2, and *AvrII* and *FseI* were used to digest pA4-653. All enzymes were acquired from New England Biolabs, Ipswich, MA. Restriction digestion was conducted on 2 mm plugs slices in 200 µl restriction mixture with 10–50 units of enzyme, at 37°C for 1 hour. Electrophoresis was conducted with the following conditions: 1.0% SKG agarose, 14°C, initial switch of 0.55 s, final switch of 5 s, 6 V/cm, for 22 h.

### Type IVB pili and plasmid screening

*pilQ* was chosen as the initial target for a population wide screening for the type IVb pilus operon because it is one of the most conserved genes found in this operon. Using PCR we screened for the presence of type IVb pili in a collection of 107 human and animal clinical isolates, representing 84 different *Salmonella* serovars (Table S2). These isolates were selected to represent multiple isolates of the serovars identified to carry the type IVb pilus operon and to represent serovar diversity. If the PCR results were positive for the presence of *pilQ*, additional PCRs targeting the *rci* and *pilV* genes were performed. For IncI1-IncFIB plasmid screening, the same 107 isolates were used. Primers were designed based on the IncI1 and IncFIB replicons, primers and PCR conditions are available in Table S6.

## Supporting Information

Figure S1 Tree generated using the Mauve algorithm with IncH1 plasmids. Alignment was generated of plasmids found in *Salmonella* serovars (i.e., Montevideo, Paratyphi A, Typhi, and Choleraesuis), *S. marcescens*, *E. coli* and *E. cloacae*.
(PDF)

Figure S2 Tree generated using the Mauve algorithm of IncI1 plasmids in E. coli and Salmonella, and IncI1-IncFIB plasmids found in this study.
(PDF)

Figure S3 Blast comparison of SGI2 and its six variants. Green arrows indicate coding regions, and regions with >50% homology are linked by grey shaded areas.
(PDF)

Figure S4 Phylogenetic tree inferred with Maximum Likelihood showing evolutionary relationships between pilQ sequences found in this study and previously reported pilQ sequences.
(PDF)

Figure S5 PFGE dendogram for the four S. Inverness isolates positive for the type IVb pilus operon and the IncI1-IncFIB cointegrated plasmid. Four different XbaI PFGE patterns were identified for these four S. Inverness isolates harboring the type IVb pilus operon and the IncI1-IncFIB replicons.
(PDF)

Table S1 Table containing plasmids, replicon types, host and accession number used for comparative analysis.
(PDF)

Table S2 Table containing list of isolates used for PCR screening, and results of PCR screening for *pilQ*, *pilV*, *rci*, IncI1, and IncFIB.
(PDF)

Table S3 List of putative genomic islands detected among the 16 genomes analyzed in this study.
(PDF)

Table S4 List of composite and unit transposons detected among the 16 genomes analyzed in this study.
(PDF)

Table S5 List of the 42 phages identified in this study that represent a size of at least 20 kb and that were identified on only one contig. Phages were classified based on homology to previously described phages in 12 groups.
(PDF)

Table S6 Table containing primers & PCR conditions used for plasmids and ICES1 validation and for population PCR-based screen.
(PDF)
