# Supplementary figures and images for: Identification and Characterization of Novel Salmonella Mobile Elements Involved in the Dissemination of Genes Linked to Virulence and Transmission
Source: PLoS One. 2012 Jul 20;7(7):e41247. doi: 10.1371/journal.pone.0041247 (PMC3401170; doi:10.1371/journal.pone.0041247)

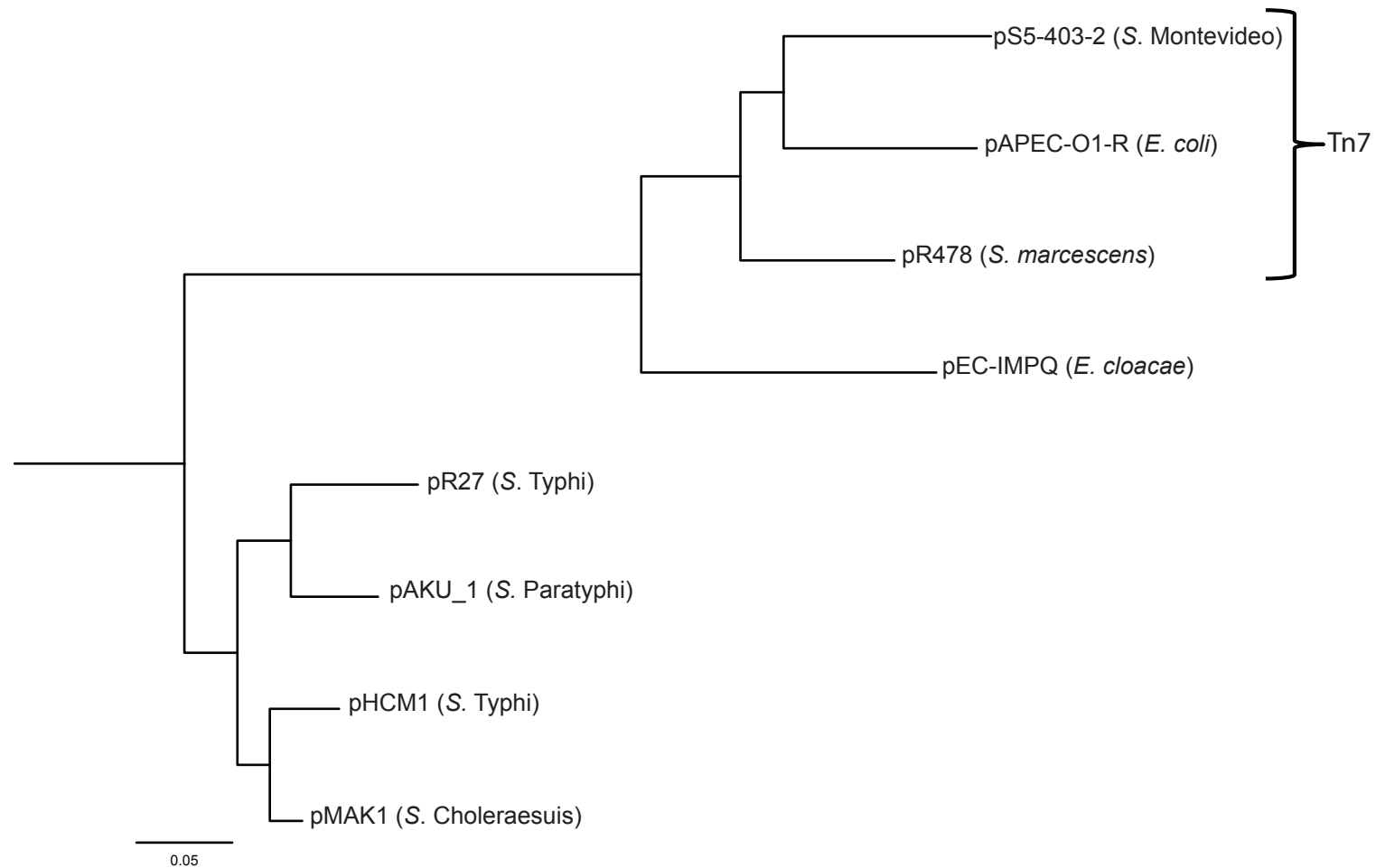

Supplement: Figure S1 — Tree generated using the Mauve algorithm with IncH1 plasmids. Alignment was generated of plasmids found in Salmonella serovars (i.e., Montevideo, Paratyphi A, Typhi, and Choleraesuis), S. marcescens, E. coli and E. cloacae. (PDF) [file pone.0041247.s001.pdf]

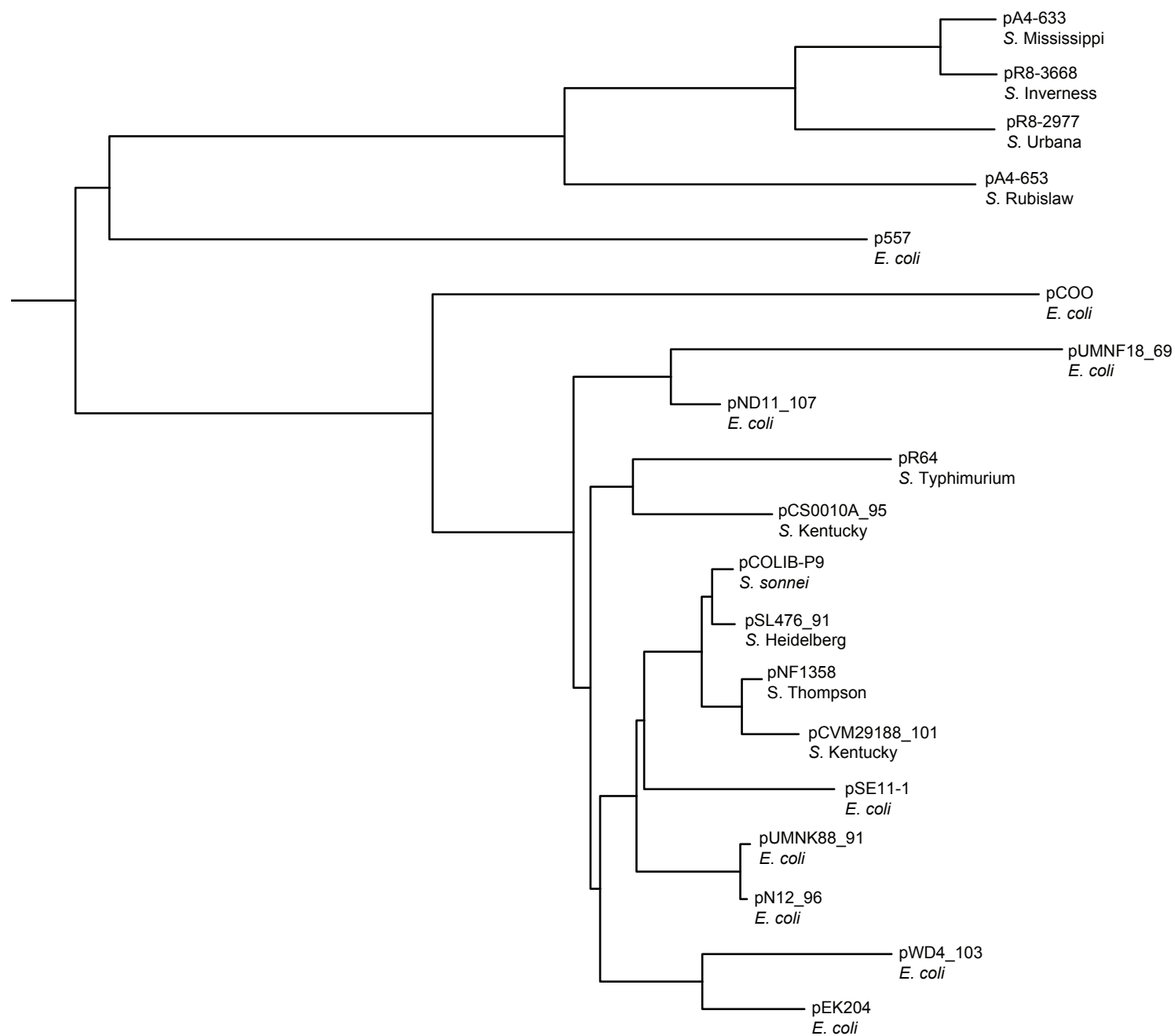

0.06

Supplement: Figure S2 — Tree generated using the Mauve algorithm of IncI1 plasmids in E. coli and Salmonella, and IncI1-IncFIB plasmids found in this study. (PDF) [file pone.0041247.s002.pdf]

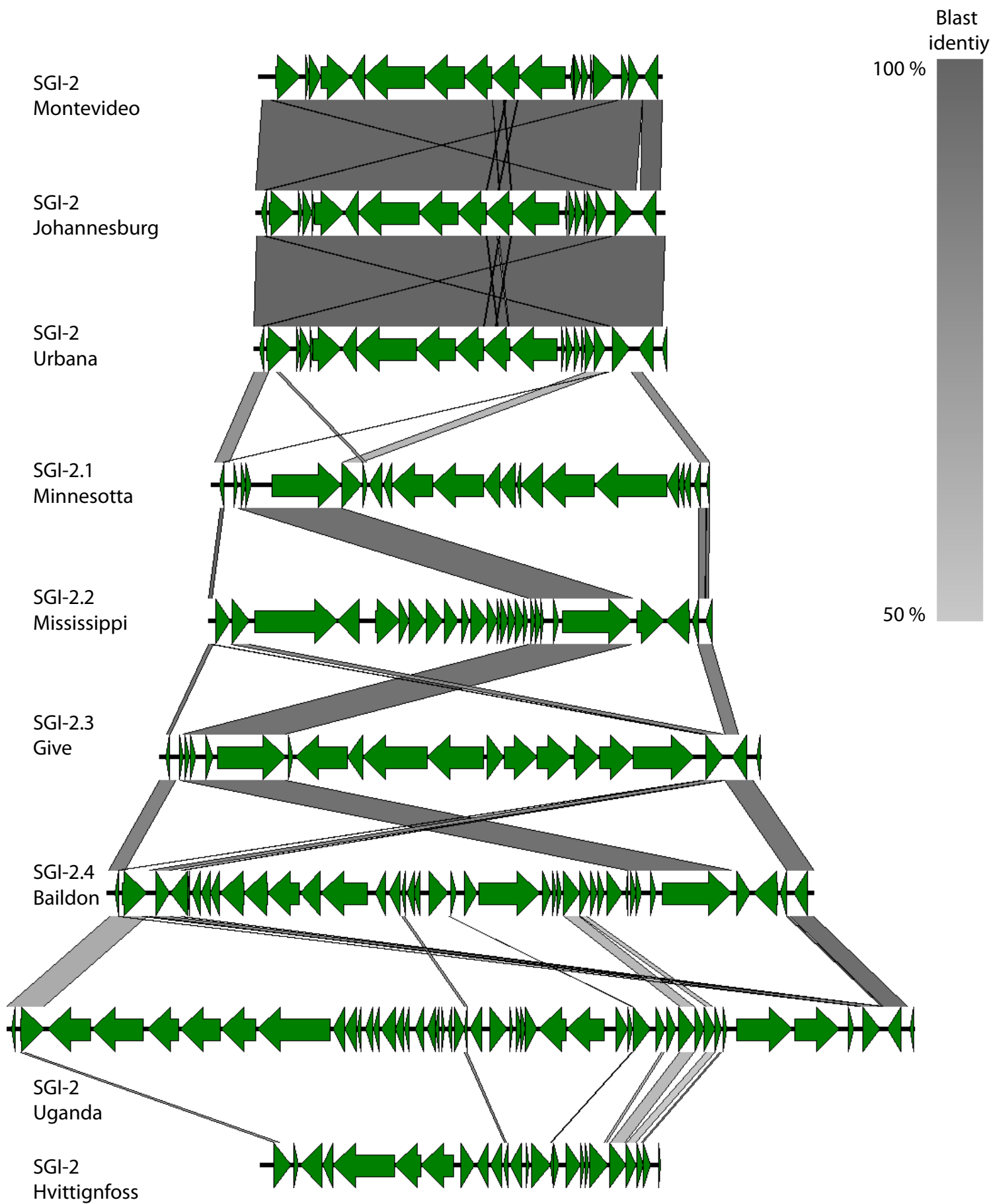

Supplement: Figure S3 — Blast comparison of SGI2 and its six variants. Green arrows indicate coding regions, and regions with >50% homology are linked by grey shaded areas. (PDF) [file pone.0041247.s003.pdf]

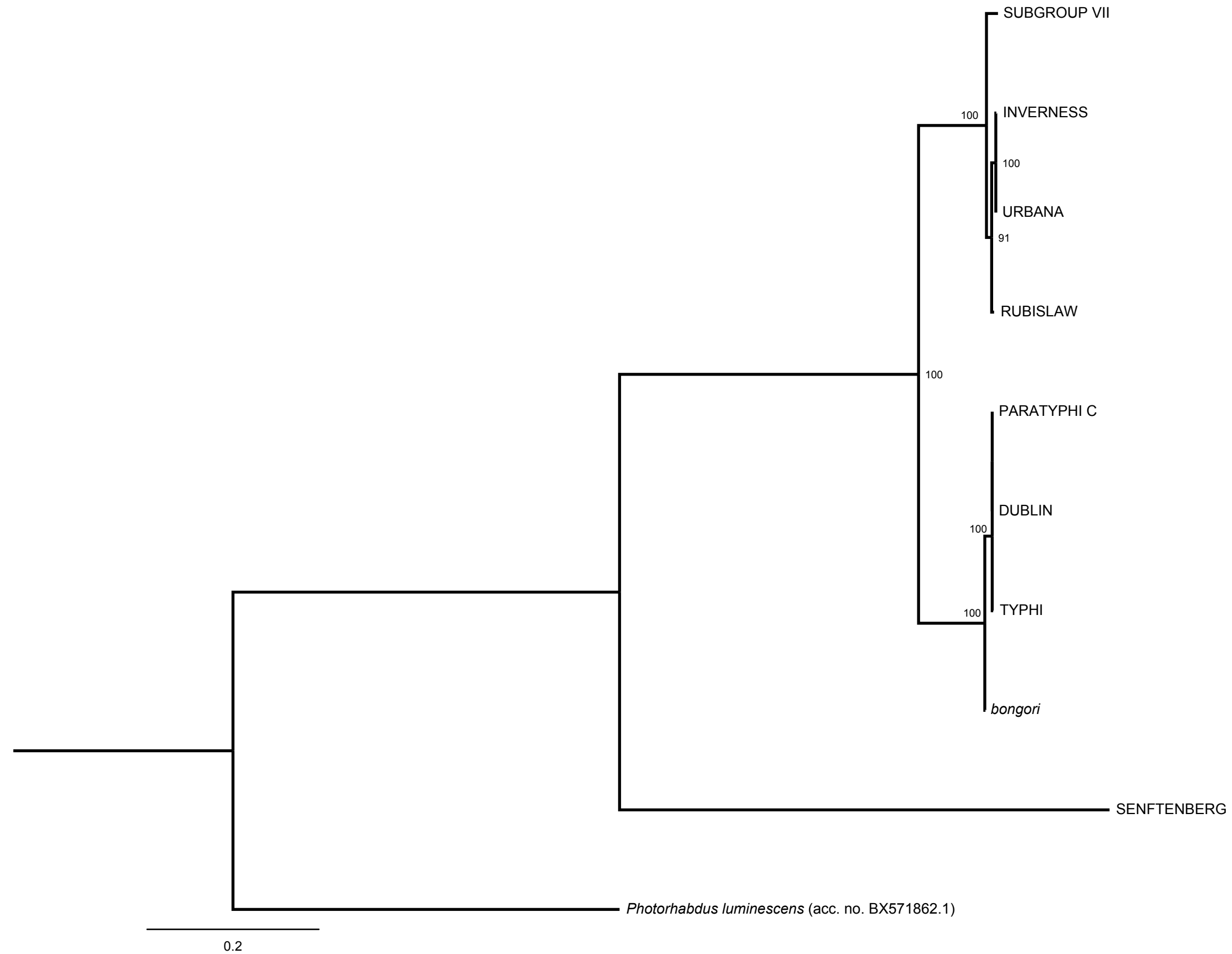

Supplement: Figure S4 — Phylogenetic tree inferred with Maximum Likelihood showing evolutionary relationships between pilQ sequences found in this study and previously reported pilQ sequences. (PDF) [file pone.0041247.s004.pdf]

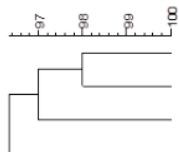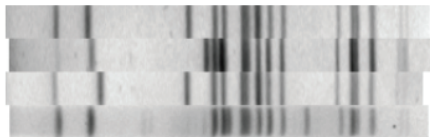

| Isolate | Isolation year |
|---------|----------------|
| R8-3669 | 2005           |
| R8-3670 | 2006           |
| R8-3668 | 2006           |
| R8-3671 | 2008           |

Supplement: Figure S5 — PFGE dendogram for the four S. Inverness isolates positive for the type IVb pilus operon and the IncI1-IncFIB cointegrated plasmid. Four different XbaI PFGE patterns were identified for these four S. Inverness isolates harboring the type IVb pilus operon and the IncI1-IncFIB replicons. (PDF) [file pone.0041247.s005.pdf]
