# Supplementary material for: Identification and Characterization of Novel Salmonella Mobile Elements Involved in the Dissemination of Genes Linked to Virulence and Transmission
Source: PLoS One. 2012 Jul 20;7(7):e41247. doi: 10.1371/journal.pone.0041247 (PMC3401170; doi:10.1371/journal.pone.0041247)
Supplement: Table S1 — Table containing plasmids, replicon types, host and accession number used for comparative analysis. (PDF) [file pone.0041247.s006.pdf]

Table S1. Plasmids and ICEs used for comparative analysis

Plasmid	host	Replicon type	Size (kb)	source	GenBank accession No.
			**Plasmid**		
pR64	*S.* Typhimurium	IncI1	120	Human	AP005147
pNF1358	*S.* Thompson	IncI1	102	Unknown[2]	DQ017661
pCVM29188_101	S. Kentucky	IncI1	101	Chicken breast	CP001121
pSL476_91	*S.* Heidelberg	IncI1	91	Human	CP001118
pCS0010A_95	*S.* Kentucky	IncI1	95	Chicken	HQ114283
pSN254	*S.* Newport	IncA/C	176	Unknown[2]	NC_009140.1
pMAK1	*S.* Choleraesuis	IncHI	208	Unknown[2]	NC_009981
pHCM1	*S.* Typhi	IncHI	218	Human	AL513383
pR27	*S.* Typhi	IncHI	180	Human	AF250878
pAKU_1	*S.* Paratyphi A	IncHI	212	Human	AM412236
pS5-403-1*	*S.* Montevideo	IncW	53	Human	AFCS00000000
pS5-403-2*	*S.* Montevideo	IncHI	299	Human	AFCS00000000
pMAK2	*S.* Dublin	IncN	61	Unknown[2]	NC_009980
p271A	*E. coli*	IncN2	35	Human	NC_015872
pEK204	*E. coli*	IncI1	93	Human	EU935740
pEC_Bactec	*E. coli*	IncI1	92	Horse	GU371927
pSE11-1	*E. coli*	IncI1	100	Human	AP009241
pND11_107	*E. coli*	IncI1	107	Pig	HQ114281
pPWD4_103	*E. coli*	IncI1	103	Pig	HQ114284
pUMNF18_69	*E. coli*	IncI1	69	Pig	CP002891
pUMNK88_IncI1	*E. coli*	IncI1	90	Pig	CP002731
pND12_96	*E. coli*	IncI1	92	Pig	HQ114282
pCoo	*E. coli*	IncI1-IncFI	98	Unknown[2]	CR942285
p557	*E. coli*	IncI1-IncFIIA	55	Human	FN822746
pAPEC-O1-R	*E. coli*	IncHI	241	Avian	NC_009838
pR8-3668[1]	*S.* Inverness	IncI1-IncFIB	121	Human	AFCO00000000
pA4-633[1]	*S.* Mississippi	IncI1-IncFIB	122	Human	AFCR00000000
pA4-653[1]	*S.* Rubislaw	IncI1-IncFIB	152	Human	AFCT00000000
pR8-2977[1]	*S.* Urbana	IncI1-IncFIB	123	Human	AFCW00000000
pColIb-P9	*S. sonnei*	IncI1	93	Unknown[2]	AB021078
pR478	*S. marcescens*	IncHI	274	Human	NC_005211
pEC-IMPQ	*E. cloacae*	IncHI	324	Human	NC_012556
			**ICE**		
ICESb2	*S. bongori*	-	109	lizard	FN669609
CTnscr94	*S.* Senftenberg	-	114	Human	FN298496
ICESe3	*S.* subgroup VII	-	104	Human	FN298495
ICES1	*S.* Inverness	-	104	Human	AFCO00000000
ICES1	*S.* Rubislaw	-	101	Human	AFCT00000000
ICES1	*S.* Urbana	-	105	Human	AFCW00000000

[1] Plasmids analyzed in this study

[2]Source not available
