# Supplementary material for: Identification and Characterization of Novel Salmonella Mobile Elements Involved in the Dissemination of Genes Linked to Virulence and Transmission
Source: PLoS One. 2012 Jul 20;7(7):e41247. doi: 10.1371/journal.pone.0041247 (PMC3401170; doi:10.1371/journal.pone.0041247)
Supplement: Table S2 — Table containing list of isolates used for PCR screening, and results of PCR screening for pilQ, pilV, rci, IncI1, and IncFIB. (PDF) [file pone.0041247.s007.pdf]

Table S2. Table containing list of isolates used for PCR screening, and results of PCR screaning for *pilQ* , *pilV* , *rci* , IncI1, and IncIFIB

Isolate	serovars	source	isolation year	PILQ	IncI1	IncIFIB	PilV	Rci
R8-3668*	Inverness	human	2005	(+)	(+)	(+)	(+)	(+)
R8-3669	Inverness	human	2006	(+)	(+)	(+)	(+)	(+)
R8-3670	Inverness	human	2006	(+)	(+)	(+)	(+)	(+)
R8-3671	Inverness	human	2008	(+)	(+)	(+)	(+)	(+)
R8-4461	Rubislaw	human (blood)	2009					
S5-477	Rubislaw	human	2004			(+)		
A4-653*	Rubislaw	human	2005	(+)	(+)	(+)	(+)	(+)
S5-388	Urbana	human	2004					
S5-410	Urbana	human	2004					
S5-659	Urbana	human	2005					
S5-660	Urbana	human	2005					
R8-2977*	Urbana	human	2008	(+)	(+)	(+)	(+)	(+)
S5-661	Urbana	human	2005	(+)			(+)	(+)
R6-535	Dublin	human	2007					
R6-607	Dublin	calf (lung)	2007					
R8-1271	Dublin	bovine (lung)	2007					
R8-1572	Dublin	human (blood)	2008					
R8-1599	Dublin	human	2008					
R8-2962	Dublin	human (blood)	2008					
R8-3349	Dublin	human	2008					
R8-3568	Dublin	human (blood)	2009					
R8-3570	Dublin	human (blood)	2009					
R8-4015	Dublin	bovine (lung)	2009					
R8-4423	Dublin	human	2009					
R8-4810	Dublin	human	2010					
S5-407	Dublin	human	2004					
S5-439	Dublin	human	2004					
S5-403**	Montevideo	human	2004	(-)	(-)	(-)	(-)	(-)
R8-153	Aarhus	human	2008					
R8-3524	Aberdeen	human (blood)	2008					
S5-469	Abony	human	2004					
S5-551	Adelaide	bovine	2004					
S5-417	Agbeni	human	2004					
R8-2924	Alachua	human	2008					
A4-650	Amager	human	2005					
S5-453	Arechavaleta	human	2004					
R6-199	Baildon	human	2006					
R8-2449	Bareilly	human (urine)	2008					
R8-1295	Barranquilla	human	2008					
R8-2917	Berta	human	2008			(+)		
A4-577	Bovismorbificans	human	2005					
R8-457	Concord	human	2008					
R8-092	Corvallis	human	2007					
R8-792	Cotham	human (urine)	2008					
S5-632	Cubana	human	2004					
A4-670	Ealing	human	2005					
S5-668	Freetown	human	2005					
R8-2934	Gaminara	human	2008					
R6-992	Georgia	human	2007					
R8-2600	Glostrup	human	2008					
A4-617	Hartford	human	2005					
R8-3386	Hindmarsh	human	2009			(+)		
R6-227	Holcomb	human	2006			(+)		
R8-789	Hvittingfoss	human	2008					
R8-091	Idikan	human (urine)	2007					
R6-527	Indiana	human	2007					
R8-3499	Johannesburg	human	2009					
R6-203	Kiambu	human	2006					
S5-712	Kintambo	human	2005					
A4-595	Kisarawe	human	2005					
R8-2447	Kottbus	human	2008					
R8-2112	Litchfield	human	2008					
R8-459	London	human	2008					
R8-3555	Luciana	human	2009					
R8-1303	Manhattan	human	2008	(+)	(+)	(+)	(+)	(+)
R8-2520	Miami	human	2008					
R6-244	Mikawasima	human	2006					
R8-2455	Mississippi	human	2008					
S5-654	Nyanza	human	2005					
R8-144	Overschie	human	2008					
R8-2486	Panama	human	2008					
R6-883	Paratyphi A	human	2007					
R6-305	Paratyphi C	human	2006	(+)		(+)		
S5-481	Pomona	human	2004					
R8-1546	Poona	human	2008					
A4-590	Putten	human	2005					
R8-3521	Remo	human	2008					
A4-827	Sandiego	human	2006					
R8-1526	Telelkebir	human	2008					
R8-3597	Tilene	human	2009					
R6-526	Wandsworth	human	2007			(+)		
A4-633*	Mississippi	Human	2005		(+)	(+)		
S5-373	Braenderup	Human	2004					
S5-395	Javiana	Human	2004					
S5-408	Stanley	Human	2004					
S5-415	Enteritidis	Human	2004			(+)		
S5-438	Weltevreden	Human	2004					
S5-447	Paratyphi B var. Ja	Human	2004					
S5-448	Heidelberg	Human	2004					
S5-451	Mbandaka	Human	2004					
S5-458	Schwarzengrund	Human	2004					
S5-480	Heidelberg	Human	2004					
S5-483	Enteritidis	Human	2004			(+)		
S5-487	Give	Human	2004					
S5-490	Worthington	Human	2004					
S5-504	Muenchen	Human	2004					
S5-517	Agona	Human	2004					
S5-536	Typhimurium	Human	2004			(+)		
S5-540	Anatum	Human	2004					
S5-543	Hadar	Human	2004					
S5-639	Newport	Human	2004					
S5-642	Oranienburg	Human	2004					
S5-648	Blockley	Human	2004					
S5-649	Saintpaul	Human	2004					
S5-658	Senftenberg	Human	2005					
S5-961	Virchow	Human	2005					
R6-542	Manhatan	animal	2007	(+)			(+)	(+)
R8-1550	Manhatan	human	2008					
R8-2473	Manhatan	human	2008					
R8-2480	Manhatan	human	2008					
R8-2498	Manhatan	human	2008					

* Sequenced in this study
** negative control
