# Supplementary material for: Identification and Characterization of Novel Salmonella Mobile Elements Involved in the Dissemination of Genes Linked to Virulence and Transmission
Source: PLoS One. 2012 Jul 20;7(7):e41247. doi: 10.1371/journal.pone.0041247 (PMC3401170; doi:10.1371/journal.pone.0041247)
Supplement: Table S3 — List of putative genomic islands detected among the 16 genomes analyzed in this study. (PDF) [file pone.0041247.s008.pdf]

Table S3. List of putative genomic islands detected among the 16 genomes analyzed in this study.

Putative element & insertion site	Similar element or some ORFs also found in:	Similar elements within the 16 genomes	Size (genome location)	Accessory genes: virulence, antimicrobial resistance & advantage genes and comments
Genomic Island (SGI2)<br>Insertion site: tRNA-Leu-CAA in all, except tRNA-SeC(p)-TCA in Hvittingfoss	Some ORFs are found in *Vibrio*, *Shewanella*, *E. coli*, Typhimurium	[1]Johannesburg<br>[1]Urbana<br>[1]Montevideo<br>[1]Uganda<br>[1]Baildon<br>[1]Give<br>[1]Mississippi<br>[1]Minnesota<br>[1]Hvittingfoss	19kb (4,572,509-4,591,562)<br>19kb (4,620,125-4,638,806)<br>19kb (4,822,925-4,841,643)<br>42kb (4,673,829- 4,716,029)<br>35kb (4,652,656- 4,687,953)<br>31kb (4,468,639- 4,500,045)<br>25kb (4,552,081- 4,576,847)<br>23kb (4,539,391- 4,562,853)<br>17kb (3,429,068-3,448,448)	-Type I Restriction modification (RM) system: Montevideo, Johannesburg, Uganda, Hvittingfoss (same RM found in all four genomes)<br>-YkfI toxin-YfjZ antitoxin in Montevideo, Johannesburg, Urbana, Baildon, Uganda, Mississippi, Hvittingfoss<br>-Type I Restriction modification system: Baildon<br>-Type II Restriction modification system: Uganda, Minnesota (same RM for these two genomes), Mississippi (RM different from Uganda & Minnesota)<br>-Type III Restriction modification system: Give<br>-Pectin lyase fold/virulence factor in Baildon<br>- Guanine nucleotide-binding proteins: Give and Uganda (different gene in these two genomes)<br>-Dynamin in Give (endocytosis related protein)<br>-Bleomycin resistance protein in Uganda<br>-Post-segregation antitoxin CcdAB: Baildon, Give, Minnesota, and Mississippi<br>-Integrase in all genomes
Genomic Island (SGI3)<br>Insertion site: GDP-mannose pyrophosphorylase gene	ORFs in *Yersinia*, *Shewanella*, *Vibrio*	[1]Mississippi	31kb (1,501,149- 1,532,251)	- OpgC (associated with survival in low osmolarity)<br>- sensory box histidine kinase/response regulator<br>-Chemotaxis regulator, anti-sigma b factor antagonist RsbV<br>-Beta-galactosidase<br>-globin-like protein<br>-transposase and integrase
Genomic Island (SGI4)<br>Insertion site: tRNA-Phe-GAA	Newport, Kentucky, Schwarzengrund, *E. coli*	[1]Mississippi<br>[1]Urbana<br>[1]Montevideo<br>[1]Minnesota<br>[1]Give<br>[1]Hvittingfoss<br>[1]Gaminara<br>[1]Johannesburg	32 kb(4,375,351-4,407,345)<br>18kb (4,457,489-4,475,243)<br>18kb (4,376,055-4,394,457)<br>17kb (4,661,369-4,678,908)<br>17kb (4,327,812-4,345,270)<br>17kb (4,424,773-4,442,320)<br>17kb (4,427,681-4,445,223)<br>17kb (4,484,388-4,501,284)	-SdiA-regulated protein in all 16 genomes<br>-Fimbriae operon in Gaminara, Minnesota, Montevideo, Hvittingfoss, Urbana, Inverness, Adelaide, Alachua, Senftenberg and Give<br>-RM system in Mississippi

		[1]Adelaide<br>[1]Alachua<br>[1]Inverness<br>[1]Baildon<br>[1]Uganda<br>[1]Wandsworth<br>[1]Rubislaw<br>[2]Senftenberg	15kb (1,974,980-1,989,993)<br>13kb (4,474,210-4,487,682)<br>9kb (4,549,011-4,558,537)<br>8kb (4,475,396-4,483,913)<br>8kb (4,522,309-4,530,674)<br>8kb (4,563,002-4,570,946)<br>6kb (4,721555-4,727,667)<br>18kb (4,724,517-4,742,586)	
Genomic Island (SGI5)<br>Insertion site: tRNA-Asn-GTT and tRNA-Ser-GGA	Orfs in *Erwinia, E. coli*	[1]Baildon<br>[1]Inverness	18kb (1,512,990 -1,530,515)<br>14kb (2,312,120-2,325,639)	-Endoprotease in both genomes<br>-Transcriptional regulator in Baildon<br>-MobA/MobL protein in Inverness and Baildon
Genomic Island SGI6)<br>Insertion site: tRNA-Asn-GTT	Paratyphi B, *Vibrio*	[1]Uganda<br>[1&4]Wansdworth	23kb (1,322,608-1,345,909)<br>21kb (2,391,030-2,412,044)	-Molybdenum cofactor biosynthesis<br>-Phage associated genes<br>- Chromosome partition protein, MukB
Genomic Island (SGI7)<br>Insertion site: tRNA-Leu-CAA	*E. coli, Citrobacter, Yersinia*	[1]Inverness	21kb (4,734,422- 4,755,415)	-Endonuclease relaxase, MobA/VirD2<br>-DNA-cytosine methyltransferase<br>-Type II restriction enzyme<br>-Magnesium transporting ATPase
Genomic Island (SGI8)<br>Insertion site: tRNA-Ser-CGA	Dublin, Gallinarum, Enteritidis, *E. coli, Yersinia*	[1]Hvittingfoss	26kb (1,485,690-1,511,984)	MobA/MobL protein, restriction modification system and Pilus proteins
Genomic Island (SGI9)<br>Insertion site: tRNA-Leu-CAA?	Typhi, Paratyphi, *E. coli, Vibrio*	[3]Senftenberg	26kb (4,895,726-4,921,938)	-PTS system, mannose-specific<br>-Helicase<br>-Transposases<br>-Post-segregation antitoxin CcdAB
Genomic Island (SGI10)<br>Insertion site: tRNA-Leu-CAA	Choleraesuis, *Yersinia, E. coli*	[1]Rubislaw	24kb (4,871,601- 4,895,743)	-Endonuclease relaxase, MobA/VirD2<br>-Arylsulfatase regulator<br>-phage associated genes

[1] one contig in scaffolds

[2] two contigs in scaffolds

[3] three contigs in scaffolds

[4]element could be longer, because contig stops in the last or first predicted ORF
