# Supplementary material for: Identification and Characterization of Novel Salmonella Mobile Elements Involved in the Dissemination of Genes Linked to Virulence and Transmission
Source: PLoS One. 2012 Jul 20;7(7):e41247. doi: 10.1371/journal.pone.0041247 (PMC3401170; doi:10.1371/journal.pone.0041247)
Supplement: Table S4 — List of composite and unit transposons detected among the 16 genomes analyzed in this study. (PDF) [file pone.0041247.s009.pdf]

Table S4. Transposons detected among the 16 genomes analyzed in this study. Insertion sequence (IS) elements were not analyzed.

Unit transposons				
**Putative element & insertion site**	**Similar transposon also found in:**	**Similar elements within the 16 genomes**	**Size (genome location)**	**Accessory genes: virulence, antimicrobial resistance & advantage genes and comments**
transposon (Tn31-like)<br>Insertion site: putative inner membrane protein (NP_459469 for Typhimurium)	Typhi, Typhimurium, Choleraesuis, Agona, *E. coli, Shigella*	[1]Johannesburg<br>[1]Hvittingfoss<br>[1]Baildon<br>[1]Senftenberg<br>[1]Inverness<br>[1]Urbana<br>[1]Give<br>[3]Minnesota<br>[1]Uganda<br>[1]Wandsworth<br>[1]Rubislaw<br>[1]Adelaide<br>[1]Mississippi<br>[1]Montevideo<br>[1]Gaminara<br>[1]Alachua	10kb (660,588-670,484)<br>10kb (746,539-756,230)<br>10kb (770,300-780,177)<br>10kb (627,476-637,375)<br>10kb (640,515-650,209)<br>10kb (586,671-596,832)<br>10kb (584,421-594,020)<br>10kb (629,383-639,593)<br>10kb (616,292-626,034)<br>10kb (630,168-639,785)<br>10kb (568,904-578,709)<br>10kb (641,317-651,190)<br>10kb (552,680-562,493)<br>8kb (571,537-579,154)<br>8kb (555,044-562,540)<br>8kb (550,764-558,758)	-Acriflavin resistance operon and potassium efflux system KefA are found in all 16 genomes<br>-Transposase is present in all the genomes, except for the Montevideo isolate. Insertion of this transposon in serovar Montevideo genome is ambiguous
transposon<br>Insertion site: tRNA(Cytosine32)	Newport, Typhimurium, Kentucky, Agona, Schwarzengrund, *E. coli, Cronobacter*	[1]Rubislaw<br>[1]Adelaide<br>[1]Alachua<br>[1]Senftenberg<br>[1]Montevideo<br>[1]Minnesota<br>[1]Johannesburg<br>[1]Urbana<br>[1]Gaminara<br>[1]Hvittingfoss<br>[1]Baildon<br>[1]Inverness<br>[1&4]Give<br>[1]Uganda<br>[1]Wandsworth<br>[1]Mississippi	5kb (1,724,305-1,729,415)<br>5kb (1,671,781-1,676,312)<br>5kb (1,633,660-1,638,266)<br>5kb (1,802,173-1,807,483)<br>5kb (1,590,956-1,596,222)<br>5kb (1,802,924-1,807,595)<br>4kb (1,809,422-1,813,067)<br>4kb(1,580,964-1,584,638)<br>2kb (1,594,888-1,596,487)<br>1kb (240,832-242,024)<br>1kb (1,885,528-1,886,517)<br>1kb (1,765,809-1,766,835)<br>1kb(1,618,787-1,619,612)<br><br>1kb (1,702,226-1,703,226)<br>1kb (1,828,134-1,829,236)<br>1kb (1,655,404-1,656,290)	-Ethidium bromide-methyl viologen resistance protein, EmrE: present in all 16 genomes<br>-Universal stress protein F is present in 15 genomes (Johannesburg, Gaminara, Minnesota, Rubislaw, Hvittingfoss, Wandsworth, Senftenberg, Urbana, Baildon, Adelaide, Alachua, Give, Inverness, Mississippi, Montevideo)<br>-Integrase is absent in Baildon, Inverness, Give, Minnesota, Uganda, Rubislaw, Adelaide, Alachua, and Mississippi<br>-Insertion site is ambiguous in Give
transposon<br>Insertion site: tRNA-	Dublin, Typhi, Typhimurium, Newport,	[1&5]Johannesburg<br>[1]Gaminara	11kb (477,314-488,374)<br>9kb (1,372,782-1,381,526)	-G-nucleotide exchange factor SopE2 (>90% identity) in all genomes

Thr-CGT, but in some genomes is ambiguous	Gallinarum	[1]Hvittingfoss<br>[1]Uganda<br>[1]Wandsworth<br>[1]Baildon<br>[1]Inverness<br>[1]Urbana<br>[1]Montevideo<br>[1]Give<br>[1]Minnesota<br>[1]Rubislaw<br>[1]Alachua (only SopE)<br>[1]Adelaide (only SopE)<br>[1]Mississippi (only SopE)	7kb (1,617,397-1,624310)<br>6kb (1,482701-1,489,126)<br>6kb (1,321,821-1,327,562)<br>6kb (1,670,728-1,676,897)<br>6kb (1,519,528-1,525,301)<br>6kb (1,358,462-1,364,101)<br>5kb (1,313,529-1,318,648)<br>5kb (1,361,295-1,366,574)<br>5kb (1,543,167-1,548,022)<br>5kb (1,491,946-1,496,812)<br>790bp (1,411,658-1,412,448)<br>768bp (1,454,377-1,455,145)<br>763bp (1,429,047-1,429,810)	-In Adelaide, Alachua and Mississippi only *sopE* and none of the other genes rest in the island<br>-Fimbriae genes: Johannesburg, Gaminara
transposon<br>Insertion site: GCN5-related N-acetyltransferase	Saintpaul, Weltevreden, Paratyphi, Virchow	[1]Minnesota<br>[1]Adelaide<br>[1]Inverness<br>[1]Uganda<br>[1]Alachua<br>[1]Senftenberg<br>[1]Wandsworth<br>[1]Montevideo<br>[1]Baildon<br>[1]Urbana<br>[1]Johannesburg<br>[1]Gaminara<br>[1]Mississippi<br>[1]Give	5kb (485,801- 490,526)<br>4kb (425,875- 429,580)<br>4kb (3,221,070- 3,224,786)<br>4kb (3,134,737-3,138,283)<br>4kb (3,031,322-3,034,875)<br>4kb (3,310,377-3,314,805)<br>3kb (3,236,697-3,239,952)<br>3kb (3,081,573-3,084,725)<br>2kb (3,001,680- 3,003,403)<br>2kb (3,017,330-3,019,182)<br>2kb (3,024,126-3,026,027)<br>2kb (2,965,849- 2,968,246)<br>2kb (3,000,985- 3,002,984)<br>2kb (2,990,804-2,992,932)	-Serine/threonine specific protein phosphatase (associated with survival in human macrophages) in all 14 genomes<br>-Located downstream of SPI-1 in Alachua, Baildon, Gaminara, Give, Inverness, Johannesburg, Mississippi, Montevideo, Senftenberg, Uganda, Urbana, Wandsworth.
Transposon<br>Putative insertion site: tRNA-Ser-GGA	Kentucky, Agona, Typhi, Paratyphi, Schwarzengrund, Typhimurium	[1]Alachua<br>[2]Urbana<br>[2]Johannesburg<br>[1]Minnesota<br>[1]Give<br>[1]Mississippi<br>[1]Hvittingfoss<br>[1]Baildon<br>[1]Rubislaw	3kb (1,235,638-1,238,280)<br>2kb (1,059,081-1,061,262)<br>1kb (1,284,389-1,285,462)<br>1kb (1,474,111-1,475,369)<br>1kb (1,589,293-1,590,551)<br>1kb (NODE_3-0.1312)<br>1kb (4,652,323-4,653,581)<br>1kb (3,321,385-3,322,643)<br>451bp (NODE_310-0.453)	-Anaerobic dimethyl sulfoxide reductase only present in Johannesburg<br>-Choline-sulfatase only present in Urbana<br>-Phosphate starvation-inducible protein PhoH only present in Alachua<br>-Integrase of the rve family
transposon<br>(IS21 family)<br>Insertion site: tRNA-Val-GAC<br>(only clear in Mississippi)	Schwarzengrund, Paratyphi, Heidelberg, Typhi, *E. coli, Shigella*	[1]Johannesburg<br>[1]Urbana<br>[1]Montevideo<br>[1]Give<br>[1]Gaminara<br>[1]Rubislaw<br>[1]Senftenberg<br>[1]Mississippi	10kb (447,932-457,854)<br>10kb (396,769-406,963)<br>10kb (392,255-402,432)<br>10kb (402,700-412,714)<br>10kb (367,026-377,025)<br>10kb (346,713-356,707)<br>10kb (444,662-454,595)<br>8kb (NODE_1679)	-Fimbriae operon and peptide transport periplasmic protein, SapA<br>-Located downstream of SPI-6 in Gaminara, Rubislaw, Senftenberg, Urbana

transposon<br>(IS3 family)<br>Insertion site:<br>Glycyl-tRNA synthetase beta chain	Choleraesuis, Enteritidis, Agona, Paratyphi, Hadar, *Enterobacter, Klebsiella, Shigella*	[1]Gaminara<br>[1]Minnesota<br>[1]Give<br>[1]Johannesburg<br>[1]Urbana<br>[1]Mississippi<br>[1]Senftenberg<br>[1]Inverness<br>[1]Uganda<br>[1]Wandsworth	5kb (3,717,736-3,722,383)<br>4kb (3,803,029-3,807,088)<br>3kb (3,756,418-3,759,258)<br>2kb (3,812,961-3,814,943)<br>2kb (3,874,210-3,876,192)<br>2kb (3,761,053-3,763,076)<br>2kb (4,101,088-4,103,102)<br>2kb (3,949,377-3,951,388)<br>2kb (3,939839-3,941,850)<br>2kb (3,930,361-3,932,372)	-Acetyltransferase
transposon<br>(Tn8-like)<br>Insertion site: Virulence protein msgA/DinI in SPI-11-like element	Typhi, Paratyphi	[1]Johannesburg<br>[1]Urbana<br>[1]Rubislaw<br>[1]Gaminara<br>[1]Minnesota<br>[1]Montevideo<br>[1]Give<br>[1]Inverness	4kb (1,239,695-1,243,954)<br>4kb (1,972,756-1,976,789)<br>4kb (2,159,425-2,163,525)<br>4kb (2,001,196-2,005,140)<br>4kb (1,063,133-1,067,187)<br>4kb (2,019,826-2,023,893)<br>4kb (2,044,614 2,048,630)<br>2kb (1,107,272-1,109,729)	-Putative pertussis-like toxin (PtIAB) and Cytolethal distending toxin subunit B in all these 8 genomes.<br>-Islet is inserted in the middle of SPI-11 in all genomes, except for Inverness (see den Bakker et al. 2011).
transposon<br>Insertion site: tRNA-Arg-TCT	Typhi, Paratyphi, Schwarzengrund, *Yersinina, E. coli, Shigella*	[2]Alachua<br>[1]Hvittingfoss<br>[1]Adelaide<br>[1]Rubislaw<br>[1]Gaminara<br>[1]Mississippi<br>[1]Senftenberg<br>[1]Montevideo	10kb (666,325-676,864)<br>7kb (833,134-840,127)<br>7kb (726,251- 733,485)<br>6kb (634,647-640,907)<br>6kb (618,660-624,448)<br>4kb (638,231- 642,580)<br>4kb (737,326- 741,788)<br>3kb (673,487- 676,305)	-O-antigen conversion and copper resistance protein present in these all 8 genomes<br>-IroB and IroC only in Alachua<br>-Integrase in Rubislaw, Adelaide, Alachua.<br>-SPI-16 associated ORFs in Adelaide, Alachua, Gaminara, Hvittingfoss, Mississippi, Senftenberg
transposon<br>Insertion site: peptide transport system permease protein, SapC	ORFs of Typhi, Paratyphi, Choleraesuis, Typhimurium *Vibrio, E. coli*	[1]Johannesburg<br>[1]Urbana<br>[1]Rubislaw<br>[1]Gaminara<br>[1]Minnesota<br>[1]Montevideo<br>[1]Give	4kb (1,765,331- 1,769,446)<br>4kb (1,534,013- 1,538,147)<br>4b (1,680,567- 1,684,397)<br>4kb (1,551,436- 1,555,535)<br>4kb (1,757,951- 1,762,074)<br>4kb (1,547,667- 1,551,160)<br>4kb (1,542,966- 1,546,310)	-Putative pertussis-like toxins (ArtAB) presents in all these 7 genomes (these toxins are present in a prophage in Inverness)
transposon (IS903)<br>Insertion site: Putative transport protein (LTSEMIS_0595)	Kentucky, Paratyphi, Typhimurium, Newport, Choleraesuis	[1]Mississippi<br>[1]Minnesota<br>[1]Alachua<br>[1]Inverness<br>[1]Wandsworth<br>[1]Senftenberg	4kb (508,551-512,622)<br>4kb (588,829-592,335)<br>4kb (508,580-512,052)<br>3kb (599,128-602,533)<br>3kb (550,921-554,416)<br>2kb (586,838-588,848)	-Tetratricopeptide repeat family protein (possible chaperons of type three secretion system)

transposon<br>Insertion site: Thiazole biosynthesis protein ThiH	Schwarzengrund, Heidelberg, Arizonae, *Dickeya*	[1]Johannesburg<br>[1]Montevideo<br>[1]Give<br>[3]Urbana<br>[1]Minnesota<br>[1]Gaminara	2kb (4,330,819-4,333,255)<br>2kb (4,498,336-4,500,743)<br>2kb (4,188,873-4,191,153)<br>2kb (4,297,069-4,299,591)<br>1kb (4,209,507-4,210,561)<br>1kb (4,262,143-4,263,298)	-Hypothetical proteins
transposon<br>Insertion site: L,D-transpeptidase, ErfK	Kentucky, Newport	[1]Gaminara<br>[1]Inverness<br>[1]Senftenberg<br>[1]Montevideo	6kb (2,133,773-2,140,013)<br>5kb (2,332,442-2,337,206)<br>5kb (2,367,220-2,372,253)<br>5kb (2,140,049-2,145,106)	-Arsenic resistance operon
Unit Transposon (Tn7-like)<br>Insertion site: NAD(FAD)-utilizing dehydrogenases	*E. coli, Enterobacter, Klebsiella*	[1]Senftenberg	37kb (1,727,559-1,764,278)	-Copper resistance<br>-Acriflavin resistance<br>-Silver resistance
transposon<br>Insertion site: tRNA-Thr-CGT	Schwarzengrund, Saintpaul, *Klebsiella*	[1]Give	5kb (431,560-436,810)	-Permease gene of the drug/metabolite transporter (DTM superfamily)
Transposon (IS1400)<br>Insertion site: tRNA-dihydrouridine synthase C	*Vibrio, Shewanella*	[1]Baildon	6kb (2,332,251-2,337,831)	-hypothetical proteins
transposon<br>Insertion site: ambiguous	Typhi, Paratyphi, Choleraesuis, Gallinarum, *E. coli*	[1]Mississippi<br>[1]Baildon<br>[1]Adelaide	9kb (379,558-388,759)<br>9kb (537,661-546,715)<br>9kb (382,581-391,371)	-Fimbriae operon present in all three genomes
transposon<br>Insertion site: ambiguous	Gallinarum	[1]Adelaide<br>[1]Uganda	3kb (431,901- 435,290)<br>3kb (453,319-456,803)	-Major facilitator superfamily<br>-Located in a prophage in Uganda
transposon<br>Insertion site: ambiguous	Arizonae, *Pantoea*	[1]Alachua	6kb (1,375,837-1,381,476)	-Secreted effector protein<br>-Ambiguous insertion site
transposon<br>Insertion site: Putative exported protein (LTSEHVI_2939)	*E. coli,* Kentucky	[1]Hvittingfoss	8kb (2,225,184-2,232,912)	-Ambiguous insertion site
transposon<br>Insertion sites: tRNA-Leu-CAA	*Nostoc*	[1]Hvittingfoss	5kb (4,609,911-4,614,766)	-Modification methylase, SinI-like<br>-Restriction endonuclease, type II
transposon<br>Insertion site: ambiguous	Agona, *E. coli*	[3]Mississippi	14kb (1,183,992-1,198,034)	-Error-prone repair protein, UmuC and cold shock protein, CspG<br>-Integrase of the rve family

transposon<br>Insertion site: ambiguous	Newport, Paratyphi, Dublin	[3]Adelaide<br>[1]Mississippi	6kb (1,421,553-1,427,532)<br>3kb (1,382,170-1,385,397)	-Acetyltransferase in both<br>-Major facilitator superfamily in Adelaide
transposon<br>Insertion site: ambiguous	*E. coli, Shigella*	[1]Senftenberg	9kb (3,747,839-3,756,634)	-Repressor of phase-1 flagellin gene
transposon<br>Insertion site: ambiguous	Newport, Typhimurium, *E. coli*	[1]Wandsworth	5kb (1,339,365-1,344,215)	-hypothetical proteins
transposon<br>Insertion site: ambiguous	Kentucky, Agona, *Pseudomonas, Klebsiella, Shewanella*	[1]Adelaide<br>[1]Senftenberg	5kb (3,262,268-3,267,212)<br>4kb (3,548,663 -3,552,546)	-Flavodoxin-like protein in Senftenberg and Adelaide<br>-Ferredoxin reductase in Senftenberg and Adelaide<br>-Heat shock protein DnaJ-like in Senftenberg
Composite transposons				
**Putative element & insertion site**	**Similar transposon also found in:**	**Similar elements within the 16 genomes**	**Size (genome location)**	**Accessory genes: virulence, antimicrobial resistance & advantage genes and comments**
Composite transposon<br>Insertion site: hypothetical protein	Similar in Dublin, Typhi, Typhimurium, Newport, Gallinarum	[1]Senftenberg	9kb (1,492,491-1,501,745)	-Secreted effector protein<br>-Zinc transporter<br>-G-nucleotide exchange factor, SopE
Composite transposon<br>Insertion site: ambiguous	Similar in Schwarzengrund, Paratyphi, Heidelberg, Typhi, *E. coli, Shigella*	[2]Uganda<br>[1]Minnesota	18kb (375,009-392,634)<br>17kb (436,514-453,149)	-Fimbriae operon and peptide transport periplasmic protein, SapA
Composite transposon<br>Insertion site: tRNA-Leu-CAA	Kentucky, *Yersinia, Serratia*	[1]Adelaide<br>[2]Gaminara	7kb (2,802,112-2,809,452)<br>7kb (4,613,484-4,620,779)	-Restriction modification system present in both genomes<br>-Prevent-host-death family protein only in Adelaide
Composite transposon (IS111A/IS1328/IS1533)<br>Insertion site: outer membrane or secreted lipoprotein	Dublin, Typhimurium, *Shewanella, Pseudomonas*	[1]Mississippi	6kb (1,762,997- 1,768,499)	-Yop effector YopM
Composite transposon (transposase 8)<br>Insertion site: ambiguous	Newport, Schwarzengrund	[1]Alachua	10kb (2,154,943-2,164,652)	-Phage associated genes
Composite transposon (transposase 20-like)	*Vibrio, Acinetobacter*	[1]Alachua	7kb (2,164,993-2,172,299)	-Restriction modification system

Insertion site: ambiguous				
Composite transposon Insertion site: tRNA-Thr-CGT	Heidelberg	[1]Baildon	15kb (566,280-581,032)	Major facilitator superfamily protein
Composite transposon Insertion site: tRNA-Arg-CCT	Kentucky	[1]Hvittingfoss	7kb (2,505,826-2,513,201)	Putative effector protein
Composite transposon Insertion site: ambiguous	Kentucky	[1]Adelaide	4kb (2,202,115-2,206,256)	-hypothetical proteins
Unit or composite transposon Insertion site: ambiguous	Agona, Schwarzengrund	[1]Senftenberg	13kb (1,153,905-1,167,464)	-Error-prone repair protein UmuD<br>-Phosphoenolpyruvate

[1] one contig in scaffolds

[2] two contigs in scaffolds

[3] Three contigs in scaffolds

[4] element could be longer, because contig stops in the last or first predicted ORF

[5] In the original scaffolds a region is inverted
