# Supplementary material for: Identification and Characterization of Novel Salmonella Mobile Elements Involved in the Dissemination of Genes Linked to Virulence and Transmission
Source: PLoS One. 2012 Jul 20;7(7):e41247. doi: 10.1371/journal.pone.0041247 (PMC3401170; doi:10.1371/journal.pone.0041247)
Supplement: Table S5 — List of the 42 phages identified in this study that represent a size of at least 20 kb and that were identified on only one contig. Phages were classified based on homology to previously described phages in 12 groups. (PDF) [file pone.0041247.s010.pdf]

Table S5. List of the 42 phages identified in this study that represent a size of at least 20 kb and that were identified on only one contig.

Homology to	Phage	*Salmonella* serovar	Morons	Size kb
PSP3	PhAde-1	Adelaide	DNA-methylase	30
	PhGam-2	Gaminara	DNA-methylase	30
	PhJoh-3	Johannesburg	-	29
	PhUga-5	Uganda	DNA-methylase	30
	PhMont-3	Montevideo	DNA-methylase	45
	PhUrb-1	Urbana	DNA-methylase	34
P22	PhJoh-2	Johannesburg	O-antigen conversion	37
	PhRub-1	Rubislaw	-	39
	PhUga-1	Uganda	O-antigen conversion	42
	PhWands-2	Wandsworth	Exopolysaccharide production, ExoZ	42
	PhMiss-1	Mississippi	-	39
	PhMont-2	Montevideo	heat shock protein GrpE	59
Gifsy-1	PhBail-1	Baildon	Virulence protein GtgA	30
	PhWands-3	Wandsworth	Virulence protein GtgA and virulence factor GipA	36
	PhHvi-1	Hvittingfoss	Virulence protein GtgA and virulence factor GipA	53
	PhInv-2	Inverness	virulence factor GipA	27
HP2	PhGiv-1	Give	-	28
	PhJoh-1	Johannesburg	-	30
	PhJoh-4	Johannesburg	DNA-methylase	30
	PhMont-1	Montevideo	DNA-methylase	30
	PhUrb-3	Urbana	DNA-methylase	31
Fels-2	PhAla-1	Alachua	DNA-methylase	36
	PhSenf-2	Senftenberg	DNA-methylase	34
	PhMont-4	Montevideo	-	33
P27	PhRub-2	Rubislaw	-	68
	PhMiss-3	Mississippi	-	23
	PhInv-1	Inverness	-	53
PhiCTX	PhRub-4	Rubislaw	DNA-methylase	34
	PhWands-1	Wandsworth	DNA-methylase and Exopolysaccharide production, ExoZ	38
	PhInv-3	Inverness	DNA-methylase	28
HK97	PhSenf-1	Senftenberg	DNA-methylase	34
	PhUgan-3	Uganda	MsgA, O-antigen conversion	29
Mu	PhAde-2	Adelaide	O-antigen conversion	41
	PhUrb-2	Urbana	-	24
Stx-2	PhGam-1	Gaminara	Secreted effector protein and attachment and invasion protein	52
ES18	PhSenf-4	Senftenberg	-	48
No homology to previously reported prophage	PhBail-2	Baildon	O-antigen conversion	21
	PhInv-1b	Inverness	DNA-methylase, Secretor effector, pertussi like toxin ArtA, ArtB	48
	PhSenf-3	Senftenberg	DNA-methylase	27
	PhRub-3	Rubislaw	Peptidase M4	54
	PhUga-2	Uganda	Drug permease	30
	PhMiss-2	Mississippi	DNA-methylase	33
