# Supplementary material for: Identification and Characterization of Novel Salmonella Mobile Elements Involved in the Dissemination of Genes Linked to Virulence and Transmission
Source: PLoS One. 2012 Jul 20;7(7):e41247. doi: 10.1371/journal.pone.0041247 (PMC3401170; doi:10.1371/journal.pone.0041247)
Supplement: Table S6 — Table containing primers & PCR conditions used for plasmids and ICES1 validation and for population PCR-based screen. (PDF) [file pone.0041247.s011.pdf]

Table S6. Table containing primers and PCR conditions used for plasmid and ICES1 validation and for population PCR-based screen.

Name	Sequence (5'-3')	PCR condition
	**Traditional PCR**	
PilQ-F	CTGCGAAAGGTGGGGTTAT	95°Cx10m, 95°Cx30s, 55°Cx30s,
PilQ-R	ATTCACGTTCTTCGGTGGTG	72°Cx45s (30 cycles), 72°Cx1m
PilV-F	GGTATTACCCGACGGTACTG	95°Cx10m, 95°Cx1m, 54°Cx1m, 72°C
PilV-R	CGACAGACACATCACCATCT	x1m (30 cycles), 72°Cx1m
Rci-F	GCTATTGAAACGGCCATGCG	95°Cx10m, 95°Cx1m, 56°Cx1m,
Rci-R	ACCCGCTCAAGTTTTCCTTCAG	72°Cx1m (30 cycles), 72°Cx1m
repFIB-F	GGGTGTTTGTTCCTACGCT	95°Cx10m, 95°Cx1m, 54°Cx1m,
repFIB-R	GTAATCCAGATAGCCAATCTCCC	72°Cx1m (30 cycles), 72°Cx1m
repIncI1-F	GACGGCCGAAGACCAGATTCGT	95°Cx10m, 95°Cx1m, 60°Cx1m,
repIncI1-R	CAGCTGGTACAGGTTCTGAATGGC	72°Cx1m (30 cycles), 72°Cx1m
pS5403-1F	GTGCAACAGCCGACGATGCC	95°Cx10m, 95°Cx30s, 60°Cx30s,
pS5403-1R	TATCCCGGAAATTTCGCAGTCGC	72°Cx1m (35 cycles), 72°Cx1m
pR8-3668-F	TTTCTCCGGGGTGAGGGGCT	95°Cx10m, 95°Cx30s, 61°Cx30s,
pR8-3668-R	ACAGATTTCGCTGACGCCGGG	72°Cx1m (35 cycles), 72°Cx1m
pA4-633-F	TGCCGCCATGCTGCGATGAT	95°Cx10m, 95°Cx30s, 61°Cx30s,
pA4-633-R	CGCGCGGCTATGTACCCAGA	72°Cx1m (35 cycles), 72°Cx1m
pR8-2977-F	GGATATCCACTGCCCGGAGCCA	95°Cx10m, 95°Cx30s, 61°Cx30s,
pR8-2977-R	GGCCGGAACGGTCACGGAAC	72°Cx1m (35 cycles), 72°Cx1m
	**Long-range PCR**	
pA4-653-F3	CGGGGGTCAGGGTTCTGGCT	94°Cx1m, 94°Cx30s, 60°Cx12m (30
pA4-653-R3	CCGGCATCTGCTGGTTACAGGGA	cycles), 72°Cx10m
pA4-653-F4	TCTGGGGGCAGGTCACGTCC	94°Cx1m, 94°Cx30s, 60°Cx12m (30
pA4-653-R4	CGGCTACAGCCCTGTGTGGC	cycles), 72°Cx10m
Hel_ssb-F	GATGCTCGATAACGACCGCTGGGATG	94°Cx1m, 94°Cx30s, 62°Cx12m (30
Hel-SsB-R	ACCTGGGAGCCCTTACGCAGA	cycles), 72°Cx10m
SsB-PilV-F	AGGATCCTGAGGTGCGTTATATGCC	94°Cx1m, 94°Cx30s, 60°Cx12m (30
SsB-PilV-R	CACAAAGTGAAGGCCATCTGCTGTC	cycles), 72°Cx10m
pS5-403-2F	GCGGAACCGGTTGGCTTCGCC	94°Cx1m, 94°Cx30s, 64°Cx4m (30
pS5-405-2R	ACACGCACGGTGGGCGCATT	cycles), 72°Cx10m
